# Vitamin D metabolic loci and preeclampsia risk in multi‐ethnic pregnant women

**DOI:** 10.14814/phy2.13468

**Published:** 2018-01-22

**Authors:** Katharyn M. Baca, Manika Govil, Joseph M. Zmuda, Hyagriv N. Simhan, Mary L. Marazita, Lisa M. Bodnar

**Affiliations:** ^1^ Department of Epidemiology Graduate School of Public Health University of Pittsburgh Pittsburgh Pennsylvania; ^2^ Center for Craniofacial and Dental Genetics Department of Oral Biology School of Dental Medicine University of Pittsburgh Pittsburgh Pennsylvania; ^3^ Department of Human Genetics Graduate School of Public Health University of Pittsburgh Pittsburgh Pennsylvania; ^4^ Department of Obstetrics, Gynecology, and Reproductive Sciences School of Medicine University of Pittsburgh Pittsburgh Pennsylvania; ^5^ Magee‐Womens Research Institute Pittsburgh Pennsylvania

**Keywords:** 1 alpha‐hydroxylase gene, preeclampsia, vitamin D, vitamin D binding protein gene, vitamin D receptor gene

## Abstract

Allelic variants in vitamin D metabolism genes may increase the risk of preeclampsia, but few studies have systematically tested this hypothesis. Our objective was to evaluate the relationship between maternal allelic variants in three vitamin D metabolism genes and risk of preeclampsia. Samples were from two case‐control studies of pregnant women who delivered in Pittsburgh, PA from 1999 to 2010 and twelve recruiting sites across the United States from 1959 to 1965. Single‐nucleotide polymorphisms (SNPs) were genotyped 50 kilobases up‐ and down‐stream in three genes (*VDR*,*GC*, and *CYP27B1*) in the samples from both studies, for a total of 744 preeclampsia cases and 2411 controls. Using multivariable logistic regression, we estimated the associations between allelic variation in each locus and preeclampsia risk by maternal race and study. Meta‐analysis was used to estimate the association across race‐study groups for each SNP. Minor allele of a noncoding region of the *VDR* gene was significantly associated with preeclampsia risk, which was verified in the meta‐analysis [odds ratio (OR), 95% confidence intervals (CI)] after adjusting for multiple comparisons [rs12831006:1.5 (1.2, 2.0), *P* < 0.0001]. The meta‐analysis identified associations for one intron *GC* variant [rs843010:1.4 (1.1, 1.9) *P* < 0.05] and two variants of the flanking region of *GC* [rs842991:1.5 (1.1, 2.0) *P* < 0.05; rs16846876:0.75 (0.58, 0.98) *P* < 0.05]. There were no statistically significant associations for *CYP27B1* SNPs. Our results provide additional support for a biological role of vitamin D in preeclampsia.

## Introduction

Preeclampsia occurs in 3–5% of all pregnancies (Ananth et al. 2013). Preeclampsia is defined by new‐onset hypertension and proteinuria, and/or any signs or symptoms of end‐organ dysfunction (renal insufficiency, impaired liver function, pulmonary edema, and thrombocytopenia) at 20 weeks of gestation or later (American: Hypertension in pregnancy, 2013). Preeclampsia is one of the leading causes of maternal and infant morbidity (Ghulmiyyah and Sibai 2012) and mortality (Duley 2009) and its complications are more likely to affect Black than White women (Lo et al. 2013).

Vitamin D may have an important role in the development of preeclampsia (Aghajafari et al. 2013). Vitamin D regulates the transcription and function of genes associated with placental invasion and normal implantation (Chan et al. 2015) and modulates antioxidant enzyme activity, inflammatory response, and blood pressure (Li et al. 2002; Versen‐Hoynck et al. 2014). However, observational studies on vitamin D deficiency and preeclampsia risk have produced mixed results (Tabesh et al. 2013; Wei et al. 2013). If vitamin D deficiency increases preeclampsia risk, then common functional sequence polymorphisms in the genes that influence vitamin D action may predispose to preeclampsia (Nilsson et al. 2004).

The *GC, CYP27B1,* and *VDR* genes are integral to vitamin D metabolism, and allelic variants in these genes have been associated with chronic diseases risk in several studies (Bailey et al. 2007; Holick et al. 2007; Jorde et al. 2012; Yang et al. 2015). However, studies in preeclampsia have been limited to only a few targeted SNPs in the *VDR* gene (Rezende et al. 2012; Zhan et al. 2015). This study further evaluated the potential associations between allelic variation in *GC, CYP27B1,* and *VDR* genes and preeclampsia risk using a systematic tagging SNP approach in two large, multi‐ethnic pregnancy cohorts.

## Materials and Methods

We used existing data and stored blood samples from two case‐control studies that have been described in detail previously: the Collaborative Perinatal Project (CPP) and the Epidemiology of Vitamin D Study (EVITA) (Bodnar et al. 2014, 2015). This study was in accordance with the ethical standards and approved by the University of Pittsburgh institutional review board. CPP enrolled more than 55,000 women at 12 U.S. medical centers from 1959 to 1965 (Niswander and Gordon 1972). From the subsample of 28,429 women with singleton pregnancies, no preexisting conditions (pregestational diabetes, hypertension, or cardiovascular disease) and a banked serum sample at <26 weeks, we selected all preeclampsia cases (*n* = 221 White, *n* = 159 Black) and a random sample of non‐preeclamptic controls among Black (*n* = 612) or White (*n* = 607) women.

Women with singleton pregnancies were eligible for EVITA if they delivered at Magee‐Womens Hospital of UPMC in Pittsburgh, Pennsylvania from 1999 to 2010 and had an available banked serum sample from aneuploidy screening at ≤20 weeks at the hospital's Center for Medical Genetics and Genomics. From the 12,861 eligible pregnancies, we selected all preeclampsia cases (*n* = 242 White, *n* = 122 Black) and randomly‐sampled non‐preeclamptic controls also among Black (*n* = 268) and White (*n* = 924) women.

Preeclampsia was defined in CPP as gestational hypertension and proteinuria of onset after 20 weeks, and return to normal in the postpartum period. Gestational hypertension was classified based on two or more measurements of systolic blood pressure ≥ 140 mm Hg and/or diastolic blood pressure ≥90 mm Hg. Proteinuria was determined by two random urine dipsticks of 1 +  protein or one dipstick of 2 +  protein which were tested for albumin every 8 weeks. At each visit, medical and obstetric events were recorded and random urine samples were tested for albumin. A validation study showed a high degree of accuracy between blood pressure and urinary albumin and original medical records (Friedman and Neff 1977). In EVITA, preeclampsia was defined by ICD‐9 codes of 642.4–642.6, indicating mild preeclampsia, severe preeclampsia/HELLP syndrome, or eclampsia. Controls were those that delivered to term and did not experience preeclampsia.

For each candidate gene, we identified tagging SNPs using the International HapMap database (phase 3) (The International HapMap Project, 2003). Tagging SNPs with minor allele frequency of 10% or more were identified for a region spanning 50 kilobases up‐ and downstream of each candidate gene using the HapMap “Utah residents with Northern and Western European ancestry” (CEU) reference population. We then supplemented this CEU specific tagging SNP panel with additional SNPs from the HapMap “Americans of African Ancestry in Southwest USA” (ASW) population in order to adequately capture variation across the candidate genes in our Black subjects. In total, 499 SNPs were selected: 39 for *CYP27B1*, 126 for *GC*, 206 for *VDR*. We also genotyped 128 ancestry informative markers in both black and white women (Kosoy et al. 2009).

Maternal serum samples in CPP were collected at ≤26 weeks of gestation and stored for 40 years at −20°C with no recorded thaws. EVITA samples were collected at ≤20 weeks of gestation and were stored at −80°C for 2 to 12 years. Serum was thawed and potential contaminants and inhibitors were removed using Qiagen kits (QIAGEN, Valencia, CA). We used REPLi‐g midi kit (QIAGEN, Valencia, CA) for whole genome amplification from the resulting serum product. QuantStudio 12K Flex platform (Life Technologies, Carlsbad, CA) was used to genotype SNPs. Genotypes were called using the TaqMan Genotyper software (Version 1.3.1, Grand Island, NY) and visual assessment of the data was used for confirmation. All plates included controls and 10% of samples were genotyped in duplicate as internal controls.

Sera from both cohorts were sent to the same Vitamin D External Quality Assessment Scheme certified laboratory. Samples were assayed for total 25‐hydroxyvitamin D (25(OH)D) [25(OH)D_2_ + 25(OH)D_3_] using liquid‐chromatography‐tandem mass spectrometry based on National Institute of Standards and Technology (NIST) standards (Holick et al. 2005). Intra‐ and the inter‐assay variations were ≤9.6% and ≤10.9%, respectively. 25(OH)D has been proven to be highly stable. No loss of 25(OH)D has been noted after leaving uncentrifuged blood as long as 72 h at 24°C, after storage of serum for years at 20°C, after exposure to ultraviolet light, or after up to 11 freeze‐thaw cycles (Zerwekh 2004). A pilot study in the CPP samples compared 25(OH)D in these serum with serum frozen for ≤2 years and found that 25(OH)D is unlikely to show significant degradation (Bodnar et al. 2009).

Potential confounding variables came from the perinatal database in EVITA and in‐person interviews for CPP. Gestational age was based on best obstetric estimate comparing menstrual dating and ultrasound estimates in EVITA and on the mother's report of the first day of her last menstrual period in CPP (ultrasound was not available in the 1960s). Both studies included data on maternal 25(OH)D concentration (nmol/l) pre‐pregnancy body mass index (<18.5, 18.5–24.9, 25–29.9, ≥30), preexisting diabetes (yes, no), maternal education (<12 years, 12 years, and >12 years), marital status (single, married), maternal age (<20, 20–29, ≥30), parity, and season of blood draw (winter (December–February), spring (March–May), summer (June–August), or fall (September–November)). Data on provider type (hospital outpatient resident clinic, hospital‐affiliated private practice) were available for women in EVITA. A composite socioeconomic status score was available for CPP, which combines education, occupation, and family income data (Niswander and Gordon 1972).

### Statistical analysis

Data‐quality control steps were performed using PLINK software (Version 1.07; Boston, MA) (Anderson et al. 2010). Samples and markers with call rates <80% were omitted from further analyses. All SNPs included in the analysis had a call rate >80%, minor allelic frequency (MAF) >0.05 and were in Hardy–Weinberg equilibrium (*P *> 0.00001).

Genetic ancestry and self‐reported race were used categorize mothers as being the African American or European (Kosoy et al. 2009). First, individual ancestral proportions were calculated and implemented in STRUCTURE 2.3 (Stanford, CA) assuming K = 1 to 4 (Hubisz et al. 2009). The number of subgroups (K = 2) was determined using an ad hoc statistic based on the rate change in the log probability of data between clusters (K) (Evanno et al. 2005). Individuals who self‐identified as White but had a > 50% probably of belonging to ASW were assigned to African American, while those with 50% or less ASW were assigned to European. All self‐reported Black mothers were assigned to African American unless they had a 100% probability of being CEU—these women were assigned to European. All models for African American mothers were adjusted for percent African ancestry.

For SNPs that passed quality control, a test of variance was conducted to determine if genotype missingness was significantly different among the four study groups (European mothers in EVITA, African American mothers in EVITA, European mothers in CPP, African American mothers in CPP) or by race, study (CPP vs. EVITA samples), or gene.

Analyses were completed on subsetted data by the four study groups, African Americans and Europeans from the CPP study on the 12 U.S medical centers from 1959 to 1965 and EVITA study on Pittsburgh mothers from 1999 to 2010. Allele frequencies by case‐control status were initially assessed for each SNP using a chi‐squared test. SNPs were further examined in logistic regression models to estimate preeclampsia odds ratios (OR) and 95% confidence intervals (CI) using the allelic model approach. Potential confounders were maternal 25(OH)D concentration, maternal age, pre‐pregnancy BMI, diabetes, smoking status, parity, education (EVITA) or socio‐economic status (CPP), sample batch number (in EVITA only), year of blood draw (in EVITA only), and study site (in CPP only). These potential confounders were chosen based on past literature on vitamin D metabolizing genes and pregnancy adverse outcomes (Swamy et al. 2011). We developed parsimonious models by removing potential confounders if their exclusion did not change the main exposure point estimate by ≥10% for associations with functional SNPs. Serum 25(OH)D concentration, mother's age, BMI, smoking status, percent African ancestry, batch number, year drawn, and site met our definition of confounding. For consistency, we included the same covariates for all tagging SNP models. All associations were adjusted for multiple comparisons and linkage disequilibrium (LD) using “LD adjusted” Bonferroni corrected *P*‐value thresholds (Nyholt 2004). LD of two SNPs characterizes dependent heritability which was measured using PLINK software.

We used random‐effects meta‐analysis to summarize estimates across the four race study groups by weighting individual coefficients by the inverse of their variance. The Higgins test (I^2^) was used to calculate heterogeneity as a measure of inconsistencies in the results of the studies. HaploReg software (version 4.1) was used to find SNPs in LD with top SNPs that were associated with preeclampsia (Ward and Kellis 2011). A sensitivity analysis on the ancestry informative markers was conducted by rerunning analysis on only self‐reported race.

## Results

**Figure 1:**
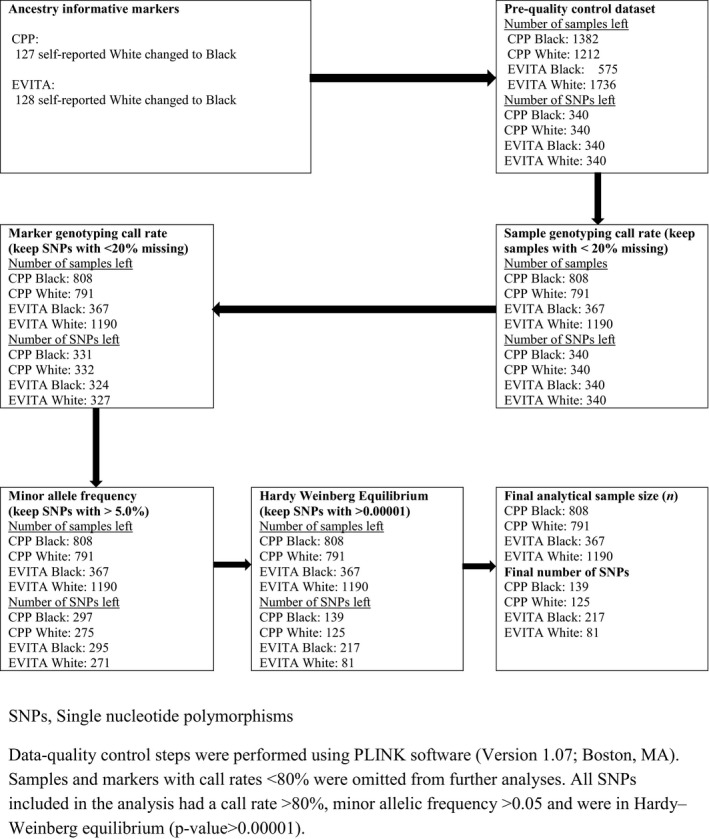
Quality control for samples from Epidemiology of Vitamin D Study (EVITA) and Collaborative Perinatal Project (CPP) and candidate single‐nucleotide polymorphisms (SNPs).

Figure 1 summarizes the final sample sizes, and final number of SNPs for each candidate gene after data quality control. Note that after quality control, there were no statistically significant differences in SNP missingness between the two racial groups (*P* = 0.09), two study samples (*P* = 0.20) or across the 4 race‐study groups (*P* = 0.12). Finally, there was no difference in missingness by chromosome (*P* = 0.29).

The mean age of women in CPP was 24 (±5.6) years compared with 29 (±6.3) years in EVITA. Compared with mothers from EVITA, more mothers in CPP were African American (52% vs. 25%), completed less education (90% vs. 32% with high school education or less), had higher prevalence of smoking (47% vs. 11%), and lower mean BMI (22.4 vs. 27.1 kg/m^2^).

In the CPP cohort, cases completed fewer years of education, smoked less, and had higher prepregnancy BMI than controls, regardless of race (Table 1). In the EVITA cohort, African American cases were more likely to be nulliparous, nonsmokers, completed less education, and obese compared with African American controls (Table 2). Except for smoking status, these differences were also present between EVITA European cases and controls.

**Table 1:** Population characteristics for controls and cases in CPP by race and preeclampsia status

	CPP: African American cases *n* = 217 Number (%)	CPP: African American controls *n* = 591 Number (%)	CPP: European cases *n* = 163 Number (%)	CPP: European controls *n* = 628 Number (%)
25(OH)D				
<25 nmol/L	57 (26)^a^	130 (22)^a^	11 (7)^a^	38 (6)^a^
25–50 nmol/L	95 (44)	284 (48)	57 (35)	214 (34)
50–75 nmol/L	41 (19)	124 (21)	51 (31)	232 (37)
≥75	24 (11)	53 (9)	44 (27)	144 (23)
Season^b^				
Winter	43 (20)	125 (21)	44 (27)	151 (24)
Spring	56 (26)	154 (26)	41 (25)	163 (26)
Summer	52 (24)	177 (30)	44 (27)	144 (23)
Fall	66 (30)	135 (23)	34 (21)	170 (27)
BMI				
<18.5	17 (8)^a^	53 (9)^a^	8 (5)^a^	57 (9)^a^
18.5–24.9	132 (61)	408 (69)	115 (70)	483 (77)
25–29.9	43 (20)	100 (17)	29 (18)	69 (11)
≥30	25 (11)	30 (5)	11 (7)	19 (3)
Socioeconomic status score				
0–20	41 (19)^a^	77 (13)^a^	10 (6)^a^	13 (2)^a^
20–39	82 (38)	207 (35)	41 (25)	95 (15)
40–59	72 (33)	213 (36)	49 (30)	188 (30)
60–79	20 (9)	71 (12)	39 (24)	188 (30)
80–100	2 (1)	23 (4)	24 (15)	144 (23)
Smoker				
Yes	78 (36)^a^	260 (44)^a^	72 (44)^a^	345 (55)^a^
No	139 (64)	331 (56)	91 (56)	283 (45)
Nulliparity				
Yes	109 (50)^a^	414 (70)^a^	81 (50)^a^	402 (64)^a^
No	108 (50)	177 (30)	82 (50)	226 (36)
Average maternal age (years)	23 ± 0.5	23 ± 0.2	24 ± 0.4	24 ± 0.2
Average gestational age of delivery (weeks)	38 ± 0.2	38 ± 0.1	39 ± 0.1	39 ± 0.1

a *P* < 0.01 for chi‐square tests comparing cases versus controls within each race.

b Winter (December–February), spring (March–May), summer (June–August), and fall (September–November).

**Table 2:** : Population characteristics for controls and cases in EVITA by race and preeclampsia status

	EVITA: African American cases *n* = 117 Number (%)	EVITA: African American controls *n* = 250 Number (%)	EVITA: European cases *n* = 248 Number (%)	EVITA: European controls *n* = 942 Number (%)
25(OH)D				
<25 nmol/L	22 (19)^a^	30 (12)^a^	5 (2)^a^	9 (1)^a^
25–50 nmol/L	47 (40)	103 (41)	30 (12)	75 (8)
50–75 nmol/L	36 (31)	85 (34)	109 (44)	377 (40)
≥75 nmol/L	12 (10)	32 (13)	104 (42)	481 (51)
Season^b^				
Winter	29 (24)	52 (21)	47 (19)	188 (20)
Spring	29 (25)	73 (29)	72 (29)	282 (30)
Summer	36 (31)	60 (24)	59 (24)	236 (25)
Fall	23 (20)	65 (26)	70 (28)	236 (25)
BMI				
<18.5	2 (2)^a^	12 (5)^a^	5 (2)^a^	19 (2)^a^
18.5–24.9	33 (28)	93 (37)	72 (29)	462 (49)
25–29.9	33 (28)	60 (24)	87 (35)	273 (29)
≥30	49 (42)	85 (34)	84 (34)	188 (20)
Education				
Some high school	19 (16)^a^	48 (19)^a^	15 (6)^a^	29 (3)
High school	50 (42)	100 (40)	57 (23)	179 (19)
Some college	30 (26)	70 (28)	55 (22)	207 (22)
College	18 (15)	32 (13)	121 (49)	527 (56)
Smoker				
Yes	11 (9)^a^	40 (16)^a^	22 (9)^a^	94 (10)^a^
No	106 (91)	210 (84)	226 (91)	848 (90)
Nulliparity				
Yes	66 (56)^a^	122 (49)^a^	156 (63)^a^	377 (40)^a^
No	51 (44)	128 (51)	92 (37)	565 (60)
Average maternal age (years)	25 ± 0.5	25 ± 0.3	31 ± 0.3	30 ± 0.1
Average gestational age of delivery (weeks)	37 ± 0.2	39 ± 0.1	37 ± 0.1	39 ± 0.02

a *P* < 0.01 for chi‐square tests comparing cases versus controls within each race.

b Winter (December–February), spring (March–May), summer (June–August), and fall (September– November).

In addition, the prevalence of women with 25(OH)D < 50 nmol/L was higher in African American mothers of CPP than EVITA (68% vs. 53%) and higher in European mothers of CPP compared with European mothers in EVITA (38% vs. 9%). The geometric means and 95% confidence intervals of 25(OH)D in African American mothers in CPP and EVITA were 36.6 nmol/L (35, 38 nmol/L**)** and 45 nmol/L (42, 48 nmol/L), respectively. These means and 95%CI of 25(OH)D in European mothers in CPP and EVITA were 54 nmol/L (52, 56 nmol/L) and 73 nmol/L (72, 74 nmol/L), respectively.

**Figure 2:**
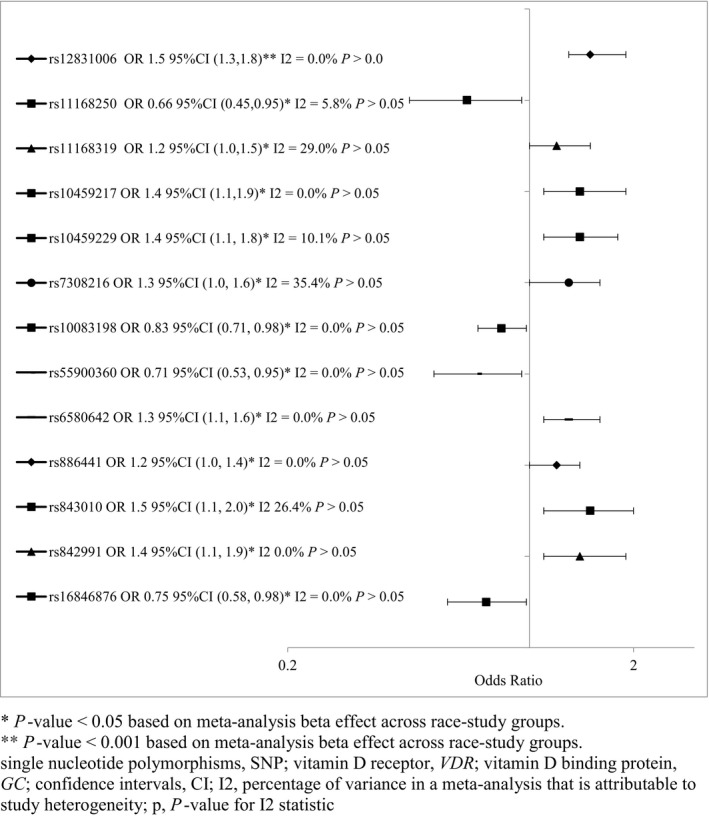
Meta‐analysis for associations between minor alleles of single‐nucleotide polymorphisms (SNPs) in *VDR* and *GC* genes and preeclampsia risk across Collaborative Perinatal Project (CPP) and Epidemiology of Vitamin D Study (EVITA) studies and maternal race groups.

Several SNPs in the noncoding and flanking regions of *VDR* were associated with preeclampsia risk in the univariate and multivariable analysis (Tables 3; Table S1). However, only two SNPs remained significant after adjustment for multiple comparisons (rs12831006, rs7300088) (Tables 3; Figure S1). Compared to the major allele, there was increased odds of preeclampsia for the T allele in the univariate (*P* < 0.05) and multivariate analyses (*P* < 0.001) which remained significant after “LD adjusted” Bonferroni correction. Among African American mothers and European mothers in EVITA, associations for the same variant attenuated and lost significance in the multivariable models. However, the meta‐analysis showed an overall increased risk of preeclampsia [OR 1.5 95%CI (1.3,1.8), *P* < 0.0001] (Fig. 2). The minor allele for rs7300088 was more commonly found in cases versus controls for European mothers in CPP (*P* < 0.05) and EVITA (*P* < 0.012). With adjustment for LD and Bonferonni, the multivariable analysis for European mothers in CPP remained significant (OR 1.8 95%CI 1.3, 2.4 *P* < 0.001), but the meta‐analysis was not significant (*P* > 0.05). The Higgins tests for the meta‐analyses were not significant, therefore the chances of false‐positives were low. The rs12831006 and rs7300088 variants were not in LD with known functional variants of *VDR*.

**Table 3:** Association between minor alleles of *VDR* variants and the risk of preeclampsia compared with major alleles by maternal race and study (Continued on Table 4).^a^

Variant name [minor/major alleles]	Study, Race	Number of controls [minor/major alleles]	Number of cases [minor/major alleles]	Univariate Analysis Odds Ratio (95% CI)		Multivariate Analysis Odds Ratio (95% CI)^b^	
rs12831006 T/A	CPP, European	194/958	71/241*	1.5	(1.1, 2.0)**	1.8	(1.3, 2.6)****
	EVITA, African American	53/425	28/198	1.1	(0.67, 1.9)	1.1	(0.67, 1.9)
	EVITA, European	291/1457	100/372*	1.3	(1.0, 1.7)**	1.6	(1.1, 2.4)
rs7300088 G/A	CPP, African American.	785/309	28/121	0.92	(0.72, 1.2)	0.88	(0.67, 1.2)
	CPP, European	252/924	85/221*	1.4	(1.1, 1.9)**	1.76	(1.3, 2.4)
	EVITA, African American	307/157	145/65	1.1	(0.78, 1.7)	2.4	(1.1, 5.5)**
	EVITA, European	346/1444	115/353	1.4	(1.1, 1.7)**	1.4	(1.0, 2.0)
rs11168250 T/G	CPP, European	251/895	43/253*	0.61	(0.43, 0.90)***	0.59	(0.40, 0.88)***
	EVITA, African American	27/445	12/210	0.94	(0.43, 2.1)	0.94	(0.43, 2.0)
rs11168319 G/A	CPP, African American	248/834	95/307	1.0	(0.79, 1.4)	1.0	(0.77, 1.4)
	CPP, European	210/938	70/242	1.3	(0.95, 1.8)	1.6	(1.2, 2.2)***
	EVITA, African American	101/375	52/160	1.2	(0.81, 1.8)	1.2	(0.81, 1.8)
	EVITA, European	304/1484	96/372	1.3	(0.98, 1.6)	1.2	(0.85, 1.8)
rs10459217 T/C	CPP, European	245/897	66/224	1.1	(0.79, 1.5)	1.4	(0.98, 1.9)
	EVITA, African American	124/328	59/145	1.1	(0.72, 1.6)	1.8	(0.89, 3.6)
rs10459229 T/C	CPP, European	240/914	76/220	1.3	(0.98, 1.8)	1.5	(1.1, 2.1)***
	EVITA, African American	123/341	60/142	1.2	(0.79, 1.7)	1.2	(0.79, 1.7)
rs7308216 G/C	CPP, African American	643/451	244/162	1.1	(0.84, 1.3)	1.0	(0.78, 1.3)
	CPP, European	260/932	77/227	1.2	(0.91, 1.6)	1.4	(1.0, 2.0)**
	EVITA, African American	254/222	133/91	1.3	(0.90, 1.8)	1.8	(0.86, 3.8)
	EVITA, European	350/1,438	111/359	1.3	(1.0, 1.6)	1.4	(0.95, 2.0)
rs10083198 T/C	CPP, African American	306/821	96/279	0.92	(0.71, 1.2)	1.0	(0.75, 1.3)
	CPP, European	108/354	190/788	0.79	(0.60, 1.0)	0.71	(0.53, 0.95)**
	EVITA, African American	161/344	53/130	0.87	(0.59, 1.3)	0.87	(0.59, 1.3)
	EVITA, European	154/516	304/1224	0.83	(0.67, 1.0)	0.77	(0.55, 1.1)
rs55900360 G/A	CPP, African American	371/1011	33/117	0.77	(0.51, 1.2)	0.73	(0.48, 1.1)
	CPP, European	276/1041	22/127	0.65	(0.41, 1.0)	0.57	(0.33, 1.0)**
	EVITA, African American	200/438	18/44	0.90	(0.49, 1.7)	0.90	0.49, 1.7)
rs6580642 T/C	CPP, African American	377/1039	49/111	1.2	(0.85, 1.7)	1.3	(0.89, 1.9)
	CPP, European	253/1012	55/178	1.2	(0.89, 1.7)	1.2	(0.82, 1.7)
	EVITA, African American	196/434	26/50	1.2	(0.69, 1.9)	1.2	(0.69, 1.9)
	EVITA, European	399/1549	73/258	1.1	(0.83, 1.5)	1.5	(1.0, 2.2)
rs886441 G/A	CPP, African American	243/700	129/350	1.1	(0.83, 1.4)	1.0	(0.79, 1.3)
	CPP, European	237/955	61/191	1.3	(0.93, 1.8)	1.3	(0.91, 1.8)
	EVITA, African American	119/282	81/154	1.2	(0.87, 1.8)	1.2	(087, 1.8)
	EVITA, European	377/1417	77/289	1.0	(0.76, 1.3)	1.4	(0.94, 2.0)

CI, confidence interval; C., B., CPP study, African American race; C., W., CPP study, European race; E., B., EVITA study, African American race*;* E., W., EVITA study, European race; *VDR,* vitamin D receptor.

*P <0.05 based on a chi‐square test. **ORs and 95% CI that reach statistical significance at *P* < 0.05. ***ORs and 95% CI that reach statistical significance at *P* < 0.01. ****ORs and 95% CI that reach statistical significance at *P* < 0.001.

a Groups not shown for each SNP did not pass quality control steps.

b Adjusted for 25(OH)D concentration, maternal age, smoking, body mass index, percent African ancestry (African American mothers only), site (CPP only), year of blood draw (EVITA only), and batch (EVITA only). Significance of the associations are given by symbols.

Several intron variants in *VDR* showed statistically significant associations only in the meta‐analysis, including 5 intron variants that were in LD with rs12831006 (rs11168250, rs11168319, rs10459217, rs7308216, and rs10459229) and four other intron variants (rs10083198, rs55900360, rs6580642, rs886441) (Fig. 2). Again, the Higgins tests for the meta‐analyses were not significant. These SNPs were not in LD with known functional variants of *VDR*.

Three SNPs in the flanking and intron regions of *GC* were associated with preeclampsia risk (Table 4; Table S2). The T allele of rs62302186 was more commonly found in cases versus controls (*P* < 0.01), and was significantly associated with increased odds of preeclampsia after adjustment for multiple comparisons (OR 1.9 95% CI 1.3, 2.7 *P* < 0.001) for European mothers in CPP. The SNP did not pass quality control for other race‐study groups. The minor alleles for rs962225 and rs962227 were also associated with increased odds of preeclampsia, and the multivariable analysis remained significant after adjustment for multiple comparisons (rs962225 OR 1.9 95% CI 1.4, 2.6 *P* < 0.001; rs962227 OR 1.7 95% CI 1.2, 2.3; *P* < 0.001). However, the meta‐analyses did not show statistical significance for either variant (*P* > 0.05).

**Table 4:** Association between minor alleles of *GC* variants and the risk of preeclampsia compared with major alleles by maternal race and study.^a^

Variant name [minor/major alleles]	Study, Race	Number of controls [minor/major alleles]	Number of cases [minor/major alleles]	Univariate Analysis Odds Ratio (95% CI)		Multivariate Analysis Odds Ratio (95%CI)^b^	
rs62302186 T/G	CPP, European	157/1011	62/236*	1.7	(1.22, 2.3)***	1.9	(1.3, 2.7)****
rs962225 G/A	CPP, European	301/733	103/157*	1.6	(1.2, 2.1)***	1.9	(1.4, 2.6)****
	EVITA, African American	244/162	116/84	0.92	(0.63, 1.3)	0.87	(0.35, 2.2)
rs962227 G/A	CPP, European	273/835	93/203*	1.4	(1.1, 1.9)**	1.7	(1.2, 2.3)***
	EVITA, African American	238/202	101/89	0.96	(0.66, 1.4)	0.93	(0.43, 2.0)
rs843010 T/C	CPP, African American	106/896	47/325	1.2	(0.85, 1.8)	1.3	(0.86, 1.9)
	CPP, European	69/1,119	26/276	1.5	(0.95, 2.4)	1.7	(1.0, 2.9)**
	EVITA, African American	62/382	18/168	0.66	(0.35, 1.2)	0.85	(0.37, 1.9)
	EVITA, European	109/1625	31/421	1.1	(0.73, 1.7)	2.0	(1.2, 3.3)***
rs842991 G/A	CPP, African American	131/947	63/321*	1.4	(1.02, 2.0)**	1.5	(1.0, 2.0)
	EVITA, African American	47/395	28/176	1.3	(0.77, 2.3)	1.3	(0.77, 2.3)
rs16846876 T/A	CPP, African American	94/944	21/311*	0.56	(0.34, 0.91)**	0.60	(0.36, 1.0)
	CPP, European	134/960	27/245	0.79	(0.51, 1.2)	0.91	(0.56, 1.5)
	EVITA, African American	44/384	20/190	0.92	(0.51, 1.6)	0.92	(0.51, 1.6)
	EVITA, European	228/1390	50/374	0.82	(0.59, 1.1)	0.62	(0.35, 1.1)

*GC*, vitamin D binding protein; CI, confidence interval; C., B., CPP study, African American race; C., W., CPP study, European race; E., B., EVITA study, African American race; E., W., EVITA study, European race.

*P <0.05 based on a chi‐square test. **ORs and 95% CI that reach statistical significance at *P* < 0.05. ***ORs and 95% CI that reach statistical significance at *P* < 0.01. ****ORs and 95% CI that reach statistical significance at *P* < 0.001.

a Groups not shown for each SNP did not pass quality control steps.

b Adjusted for 25(OH)D concentration, maternal age, smoking, body mass index, percent African ancestry *(*African American mothers only), site (CPP only), year of blood draw (EVITA only), and batch (EVITA only). Significance of the associations are given by symbols.

The meta‐analysis on *GC* SNPs did reveal significant associations for one intron variant (rs843010) and two variants of the flanking region of GC (rs842991 and rs16846876). The minor alleles for rs843010 and rs842991 were associated with increased odds of preeclampsia (rs843010 OR 1.4 95% CI 1.1, 1.9 *P* < 0.05; rs842991 1.5 95% CI 1.1, 2.0 *P* < 0.05), and rs16846876 was associated with decreased preeclampsia risk (OR 0.75 95%CI 0.58, 0.98 *P* < 0.05). The Higgins tests for the meta‐analyses were not significant. These SNPs were not in LD with SNPs of known function.

The univariate, multivariate, and meta‐analysis did not show any statistically significant associations for *CYP27B1* SNPs (Table S3). The sensitivity analysis showed no differences in the results for *VDR*,* GC*, and *CYP27B1* variants (data not shown) when using just self‐reported maternal race.

## Discussion

In the multivariate and univariate analyses, two SNPs in the flanking and noncoding regions SNPs in *VDR* and three SNPs in the flanking and noncoding regions in *GC* were associated with preeclampsia risk. Through meta‐analysis across study groups, associations were confirmed for rs12831006 *VDR* SNP. The meta‐analytic approach also revealed relationships between SNPs in the flanking and noncoding regions of *GC* and *VDR* with preeclampsia risk. However, meta‐analyses did not yield significant results for *CYP27B1* SNPs.

There has been one GWAS on preeclampsia, which observed two nonfunctional SNP associations in an intron region less than 15 kb downstream from the 3′ terminus of the Inhibin, beta B (INHBB) gene (Johnson et al. 2012). The study genotyped 538 preeclampsia cases and 540 normal pregnancy controls who were Caucasian and from Australia. Our study used the candidate gene approach using vitamin D metabolism genes due to the observed relationship between vitamin D deficiency and increased preeclampsia risk. This study approach allowed us to focus on SNPs of interest across two diverse studies. Previously, there have been few genetic association studies of vitamin D‐related genes and risk of preeclampsia, and gene coverage in two of these studies was limited to only three variants in *VDR* (rs2228570, rs731236, rs1544410) (Rezende et al. 2012; Zhan et al. 2015). In a cohort of 164 preeclamptics, 154 gestational diabetics, and 213 pregnant women without either condition, there were no case–control differences in the three *VDR* SNPs by maternal genotype, allelic, and haplotype frequencies across outcome groups (Rezende et al. 2012). In agreement with that study, we did not observe an association for *VDR* SNPs rs731236 or rs1544410 in the meta‐analysis. The *VDR* SNP rs2228570 did not pass quality control steps in our study. Unlike our study, in a Chinese population minor allele of *VDR* SNP rs1544410 was associated with an increased risk of preeclampsia (OR 1.4, 95% CI 1.1,1.6) (Zhan et al. 2015). Our inability to find a statistically significant association with rs1544410 and preeclampsia may have been due to differences in ancestral backgrounds between studies. Previous studies did not use a tagging SNP approach and consequently may have missed an underlying association with preeclampsia. We cannot compare our results on *GC* and *CYP27B1* or other SNPs within the *VDR* region because they were not measured in previous reports.

The vitamin D hormone 1,25(OH)_2_D_3_ exerts its biological activity through binding to a high‐affinity receptor (*VDR*) that acts as a ligand‐activated gene transcription factor (Pike and Meyer 2010). Genetic variation in the *VDR* gene may enhance or reduce its mRNA expression, 1,25(OH)_2_D_3_ binding affinity, or ability to transactivate target genes (Ogunkolade et al. 2002). Cell culture studies have observed possible functions of *VDR* that may influence the development of preeclampsia by maintaining an inflammatory response (Tamblyn et al. 2015), placental implantations in early pregnancy (Uitterlinden et al. 2004), and endothelial repair (Versen‐Hoynck et al. 2014). Cell culture studies have also revealed that, through a *VDR*‐mediated mechanism, 1,25(OH)_2_D_3_ suppresses renin transcription, which is important in the regulation of blood pressure (Li et al. 2002).

Past studies of nonpregnant adults observed common variations in the *GC* gene modulate vitamin D binding protein concentrations by affecting the GC protein's affinity to 25(OH)D, which in turn controls the bioavailability of free 25(OH)D (Speeckaert et al. 2006). Poor GC protein‐25(OH)D binding may reduce the serum concentration of 25(OH)D and other vitamin D metabolites(Bouillon 2011), therefore genetic variation in the gene may increase the risk of preeclampsia by increasing risk of vitamin D deficiency. Instead of measuring the concentration of GC protein, we genotyped common SNPs in *GC* that encodes the vitamin D protein.

Most of the SNPs genotyped in *VDR* and *GC* regions in this study were in the noncoding or flanking sequences of the genes. The tagging SNPs that were associated with preeclampsia in our study were not in linkage disequilibrium with known functional variants. However, they may be in linkage disequilibrium with unknown functional variants, or it is possible the associations may have occurred by chance.

We reduced the likelihood of spurious data due to failed assays and poor quality samples by setting thresholds for missing data by SNP and by samples in the quality control steps. If these steps were avoided, differences in DNA quality could have biased towards one genotype or another (Wellcome, 2007). After quality control steps, there were no differences in missingness of SNP data by race, study‐race group, or chromosome. Nonetheless, future genome wide association studies are needed to identify additional genes and alleles for preeclampsia risk. Although the measured confounders available for the CPP and EVITA study populations differed somewhat, our meta‐analytic approach allowed us to combine results to account for this limitation. Vitamin D supplement use, diet, and sunlight exposure influences the amount of pre‐vitamin D in the body which were not measured in our study. Our findings may not be generalizable to other racial groups. Our study also lacked data on fetal genotype and the ability to study maternal‐fetal genotype interaction, which may be important to adverse birth outcomes. Major strengths of our study included the large number of African American and European cases of preeclampsia and genotyping for ancestral markers rather than reliance on self‐reported race. In addition, we used a tagging SNP approach to more comprehensively assess candidate gene variation than previous studies.

If our results linking SNPs in *VDR* and *GC* with preeclampsia are confirmed, then allelic variation may have an important role in the relationship between vitamin D and preeclampsia. Further research is needed to identify the functionality of *VDR* and *GC* SNPs, to test interactions and associations between 25(OH)D and these allelic variants, to test SNP‐SNP epistatic effects, and to identify if SNPs in other vitamin D related genes have a relationship with preeclampsia risk.

## **Conflict of Interest**

The author reports no conflicts of interest in this work.

## Data Accessibility

## Supporting information




**Figure S1:** *P*‐values by base pairs in VDR, CYP27B1, and GC gene with corresponding significant *P*‐value thresholds, after Bonferroni correction and linkage disequilibrium adjustment.

**Table S1:** Association between minor alleles of *VDR* variants and the risk of preeclampsia compared with major alleles by maternal race and study.^a^
**Table S2:** Association between minor alleles of *GC* variants and the risk of preeclampsia compared with major alleles by maternal race and study.^a^
**Table S3:** Association between minor alleles of *CYP27B1* variants and the risk of preeclampsia compared with major alleles by maternal race and study.^a^
