# Supplementary material for: Vitamin D metabolic loci and preeclampsia risk in multi‐ethnic pregnant women
Source: Physiol Rep. 2018 Jan 22;6(2):e13468. doi: 10.14814/phy2.13468 (PMC5789712; doi:10.14814/phy2.13468)
Supplement: Supplementary file 1 — Figure S1: P‐values by base pairs in VDR, CYP27B1, and GC gene with corresponding significant P‐value thresholds, after Bonferroni correction and linkage disequilibrium adjustment. [file PHY2-6-e13468-s001.docx]

Supplementary Figure 1: P-values by base pairs in VDR, CYP27B1, and GC gene with corresponding significant p-value thresholds, after Bonferroni correction and linkage disequilibrium adjustment.

VDR gene

VDR p-value threshold

CYP27B1 gene

CYP27B1 p-value threshold

GC gene

GC p-value threshold

Base pairs by gene
