# Supplementary material for: Vitamin D metabolic loci and preeclampsia risk in multi‐ethnic pregnant women
Source: Physiol Rep. 2018 Jan 22;6(2):e13468. doi: 10.14814/phy2.13468 (PMC5789712; doi:10.14814/phy2.13468)
Supplement: Supplementary file 2 — Table S1: Association between minor alleles of VDR variants and the risk of preeclampsia compared with major alleles by maternal race and study.a Table S2: Association between minor alleles of GC variants and the risk of preeclampsia compared with major alleles by maternal race and study.a Table S3: Association between minor alleles of CYP27B1 variants and the risk of preeclampsia compared with major alleles by maternal race and study.a [file PHY2-6-e13468-s002.docx]

Supplemental Table 1: Association between minor alleles of *VDR* variants and the risk of preeclampsia compared with major alleles by maternal race and study.^a^

| Variant name | Study, Race | Number of controls [minor/major alleles] | | Number of cases [minor/major alleles] | Univariate Analysis Odds Ratio (95%CI) | | | Multivariate Analysis  Odds Ratio (95%CI) ^b^ | | | |
| --- | --- | --- | --- | --- | --- | --- | --- | --- | --- | --- | --- |
| rs10083198 | C., B. | 306/821 | 96/279 | | 0.92 | (0.71, | 1.2) | | 1.0 | (0.75, | 1.3) |
| C/T | C., W. | 108/354 | 190/788 | | 0.79 | (0.60, | 1.0) | | 0.71 | (0.53, | 0.95)** |
|  | E., B. | 161/344 | 53/130 | | 0.87 | (0.59, | 1.3) | | 0.87 | (0.59, | 1.3) |
|  | E., W. | 154/516 | 304/1224 | | 0.83 | (0.67, | 1.0) | | 0.77 | (0.55, | 1.1) |
| rs10459217 | C., W. | 224/897 | 66/245 | | 1.1 | (0.79, | 1.5) | | 1.4 | (0.98, | 1.9) |
| T/C | E., B. | 145/328 | 59/124 | | 1.1 | (0.72, | 1.6) | | 1.8 | (0.89, | 3.6) |
| rs10459229 | C., W. | 220/914 | 76/240 | | 1.3 | (0.98, | 1.8) | | 1.5 | (1.1, | 2.1)*** |
| C/T | E., B. | 142/341 | 60/123 | | 1.2 | (0.79, | 1.7) | | 1.2 | (0.79, | 1.7) |
| rs10747524  T/C | E., B. | 124/264 | 88/172 | | 1.1 | (0.74, | 1.6) | | 1.0 | (0.32, | 3.4) |
| rs10747527 | C., B. | 385/1007 | 29/97 | | 0.78 | (0.51, | 1.2) | | 0.82 | (0.52, | 1.3) |
| C/T | E., B. | 193/418 | 21/38 | | 1.2 | (0.65, | 2.2) | | 1.2 | (0.65, | 2.2) |
| rs10783218 | C., B. | 318/856 | 80/180 | | 1.2 | (0.89, | 1.6) | | 1.2 | (0.87, | 1.6) |
| G/A | E., B. | 167/353 | 35/79 | | 0.94 | (0.58, | 1.5) | | 0.58 | (0.30, | 1.1) |
|  | E., W. | 430/1621 | 40/123 | | 1.2 | (0.84, | 1.8) | | 1.7 | (0.98, | 2.8) |
| rs10783222 | C., B. | 257/685 | 131/403 | | 0.87 | (0.68, | 1.1) | | 0.90 | (0.70, | 1.2) |
| G/C | C., W. | 161/623 | 143/539 | | 1.0 | (0.80, | 1.3) | | 0.96 | (0.73, | 1.3) |
|  | E., B. | 137/293 | 73/171 | | 0.91 | (0.62, | 1.3) | | 1.0 | (0.50, | 2.0) |
|  | E., W. | 246/881 | 222/851 | | 0.93 | (0.76, | 1.2) | | 1.3 | (0.92, | 1.8) |
| rs10875693 | C., B. | 340/930 | 60/164 | | 1.0 | (0.73, | 1.4) | | 0.99 | (0.70, | 1.4) |
| T/A | E., B. | 179/366 | 37/78 | | 0.97 | (0.60, | 1.6) | | 0.62 | (0.34, | 1.1) |
| rs10875694 | C., B. | 359/931 | 49/159 | | 0.80 | (0.57, | 1.1) | | 0.83 | (0.58, | 1.2) |
| T/A | E., B. | 185/415 | 39/51 | | 1.7 | (1.1, | 2.8)** | | 1.3 | (0.64, | 2.6) |
|  | E., W. | 376/1382 | 82/296 | | 1.0 | (0.78, | 1.3) | | 0.86 | (0.57, | 1.3) |
| rs10875695 | C., B. | 193/561 | 197/529 | | 1.1 | (0.86, | 1.4) | | 1.1 | (0.84, | 1.4) |
| C/A | C., W. | 201/861 | 99/317* | | 1.3 | (1.0, | 1.8)** | | 1.5 | (1.1, | 2.1)*** |
|  | E., B. | 98/239 | 98/209 | | 1.1 | (0.78, | 1.7) | | 0.62 | (0.33, | 1.2) |
|  | E., W. | 324/1307 | 138/453 | | 1.2 | (0.98, | 1.5) | | 1.2 | (0.83, | 1.6) |
| rs111336890 | C., B. | 3161/996 | 5/134 | | 1.1 | (0.74, | 1.5) | | 1.0 | (0.71, | 1.4) |
| T/C | E., B. | 194/410 | 22/48 | | 0.97 | (0.55, | 1.7) | | 1.5 | (0.45, | 5.1) |
| rs11168248 | C., B. | 333/903 | 75/191 | | 1.1 | (0.79, | 1.4) | | 1.1 | (0.84, | 1.5) |
| T/G | C., W. | 260/999 | 46/177 | | 1.0 | (0.70, | 1.4) | | 1.0 | (0.68, | 1.5) |
|  | E., B. | 182/376 | 34/80 | | 0.88 | (0.55, | 1.4) | | 0.52 | (0.17, | 1.5) |
| rs11168249 | C., B. | 242/652 | 130/382 | | 0.92 | (0.72, | 1.2) | | 0.97 | (0.75, | 1.3) |
| C/T | E., B. | 140/272 | 32/148* | | 0.68 | (0.46, | 1.0) | | 0.68 | (0.46, | 1.0) |
| rs11168250 | C., W. | 253/895 | 43/251* | | 0.61 | (0.43, | 0.86)*** | | 0.59 | (0.40, | 0.88)*** |
| G/T | E., B. | 210/445 | 12/27 | | 0.94 | (0.43, | 2.1) | | 0.94 | (0.43, | 2.1) |
| rs11168256 A/G | E., B. | 204/444 | 16/26 | | 1.3 | (0.68, | 2.6) | | 1.3 | (0.68, | 2.6) |
| rs11168261 | C., B. | 361/995 | 37/115 | | 0.89 | (0.60, | 1.3) | | 0.83 | (0.55, | 1.3) |
| C/T | E., B. | 202/429 | 18/43 | | 0.89 | (0.46, | 1.7) | | 0.89 | (0.46, | 1.7) |
|  | E., W. | 371/1483 | 71/263 | | 1.1 | (0.81, | 1.4) | | 1.2 | (0.78, | 1.9) |
| rs11168264 A/G | E., B. | 173/373 | 31/69 | | 0.97 | (0.57, | 1.6) | | 0.97 | (0.57, | 1.6) |
| rs11168268 A/G | E., B. | 129/278 | 75/144 | | 1.1 | (0.76, | 1.7) | | 1.1 | (0.76, | 1.7) |
| rs11168275 | C., B. | 270/745 | 132/335 | | 1.1 | (0.85, | 1.4) | | 1.1 | (0.83, | 1.4) |
| T/C | E., B. | 144/297 | 70/167 | | 0.86 | (0.59, | 1.3) | | 1.6 | (0.92, | 2.9) |
|  | E., W. | 348/1402 | 112/364 | | 1.2 | (0.97, | 1.6) | | 1.2 | (0.80, | 1.7) |
| rs11168280 T/G | E., B. | 193/409 | 25/41 | | 1.3 | (0.72, | 2.3) | | 4.8 | (1.2, | 18.9)** |
| rs11168283 | C., B. | 323/895 | 71/159 | | 1.2 | (0.91, | 1.7) | | 1.3 | (0.95, | 1.8) |
| C/T | E., B. | 180/368 | 34/60 | | 1.2 | (0.73, | 1.8) | | 1.2 | (0.73, | 1.8) |
| rs11168286 A/G | C., W. | 174/633 | 100/367 | | 0.99 | (0.75, | 1.3) | | 0.98 | (0.72, | 1.3) |
| rs11168287 | C., W. | 143/558 | 151/546 | | 1.1 | (0.83, | 1.4) | | 1.2 | (0.89, | 1.6) |
| A/G | E., B. | 144/328 | 70/124 | | 1.3 | (0.87, | 1.9) | | 1.3 | (0.87, | 1.9) |
|  | E., W. | 201/876 | 227/772* | | 1.3 | (1.0, | 1.6)** | | 0.86 | (0.63, | 1.2) |
| rs11168293 | C., B. | 354/960 | 50/132 | | 1.0 | (0.73, | 1.5) | | 1.2 | (0.80, | 1.7) |
| G/T | C., W. | 224/789 | 76/323 | | 0.83 | (0.62, | 1.1) | | 0.71 | (0.51, | 0.98)** |
|  | E., B. | 190/399 | 24/39 | | 1.3 | (0.72, | 2.3) | | 1.3 | (0.72, | 2.3) |
|  | E., W. | 325/1177 | 113/451 | | 0.91 | (0.71, | 1.2) | | 1.1 | (0.71, | 1.6) |
| rs11168306  G/A | E., W. | 460/1678 | 26/106 | | 0.89 | (0.58, | 1.4) | | 1.0 | (0.56, | 1.8) |
| rs11168318 G/A | E., B. | 116/274 | 76/160 | | 1.1 | (0.76, | 1.7) | | 2.1 | (0.99, | 4.6) |
| rs11168319 | C., B. | 307/834 | 95/248 | | 1.0 | (0.79, | 1.4) | | 1.0 | (0.77, | 1.4) |
| A/G | C., W. | 242/938 | 70/210 | | 1.3 | (0.95, | 1.8) | | 1.6 | (1.2, | 2.2)*** |
|  | E., B. | 160/375 | 52/101 | | 1.2 | (0.81, | 1.8) | | 1.2 | (0.81, | 1.8) |
|  | E., W. | 372/1484 | 96/304 | | 1.3 | (0.98, | 1.6) | | 1.2 | (0.85, | 1.8) |
| rs11168325 | E., B. | 144/288 | 48/122 | | 0.79 | (0.52, | 1.2) | | 0.79 | (0.52 | 1.2) |
| A/C | E., W. | 197/718 | 201/794 | | 0.92 | (0.74, | 1.2) | | 0.91 | (0.65, | 1.3) |
| rs11168327 | C., W. | 215/857 | 77/309 | | 0.99 | (0.74, | 1.3) | | 1.3 | (0.93, | 1.7) |
| C/T | E., B. | 91/224 | 115/232 | | 1.2 | (0.85, | 1.7) | | 1.7 | (0.81, | 3.5) |
|  | E., W. | 342/1320 | 116/412 | | 1.1 | (0.86, | 1.4) | | 0.87 | (0.61, | 1.2) |
| rs114088610 C/G | E., B. | 187/397 | 23/71 | | 0.69 | (0.39, | 1.2) | | 0.69 | (0.39, | 1.2) |
| rs11574042 | C., B. | 317/846 | 67/208 | | 0.86 | (0.63, | 1.2) | | 0.85 | (0.62, | 1.2) |
| C/G | C., W. | 260/1004 | 42/166 | | 0.98 | (0.68 | 1.4) | | 0.99 | (0.67, | 1.5) |
|  | E., B. | 151/354 | 57/102 | | 1.3 | (0.87, | 2.0) | | 1.3 | (0.87, | 2.0) |
| rs11574044 | C., B. | 309/812 | 91/212 | | 1.1 | (0.85, | 1.5) | | 1.1 | (0.85, | 1.5) |
| A/C | C., W. | 243/965 | 37/151 | | 0.97 | (0.66, | 1.4) | | 0.96 | (0.64, | 1.4) |
|  | E., B. | 164/345 | 36/95 | | 0.80 | (0.51, | 1.3) | | 0.80 | (0.51, | 1.3) |
|  | E., W. | 386/1506 | 58/208 | | 1.1 | (0.80, | 1.5) | | 1.6 | (1.1, | 2.5)** |
| rs11574113 | C., B. | 381/1056 | 41/94 | | 1.2 | (0.82, | 1.8) | | 1.3 | (0.89, | 2.0) |
| C/G | C., W. | 283/1085 | 33/129 | | 0.98 | (0.65, | 1.5) | | 1.1 | (0.75, | 1.8) |
|  | E., B. | 214/439 | 16/33 | | 0.99 | (0.54, | 1.8) | | 0.99 | (0.54, | 1.8) |
|  | E., W. | 443/1625 | 35/183 | | 0.70 | (0.48, | 1.0) | | 0.74 | (0.44, | 1.2) |
| rs11574114 | C., B. | 370/982 | 44/120 | | 0.97 | (0.68, | 1.4) | | 0.94 | (0.64, | 1.4) |
| C/T | E., B. | 190/409 | 18/39 | | 0.99 | (0.53, | 1.9) | | 0.99 | (0.53, | 1.8) |
| rs11574143 | C., B. | 377/1041 | 33/77 | | 1.2 | (0.77, | 1.8) | | 1.3 | (0.85, | 2.0) |
| C/T | E., B. | 209/433 | 19/25 | | 1.6 | (0.82, | 3.0) | | 1.6 | (0.82, | 3.0) |
|  | E., W. | 430/1603 | 38/165 | | 0.86 | (0.59, | 1.2) | | 0.86 | (0.48, | 1.5) |
| rs11608702 | C., B. | 302/823 | 104/267 | | 1.1 | (0.82, | 1.4) | | 1.1 | (0.83, | 1.5) |
| A/T | C., W. | 200/776 | 92/384 | | 0.93 | (0.71, | 1.2) | | 0.91 | (0.67, | 1.2) |
|  | E., B. | 167/358 | 51/122 | | 0.90 | (0.59, | 1.4) | | 0.90 | (0.59, | 1.4) |
|  | E., W. | 314/1160 | 152/520 | | 1.1 | (0.87, | 1.4) | | 0.81 | (0.57, | 1.2) |
| rs11831883 | C., B. | 310/835 | 82/235 | | 0.94 | (0.71, | 1.3) | | 0.97 | (0.73, | 1.3) |
| C/T | E., B. | 157/348 | 55/86 | | 1.4 | (0.93, | 2.2) | | 1.4 | (0.93, | 2.2) |
| rs11831940 | C., B. | 267/667 | 123/349 | | 0.88 | (0.69, | 1.1) | | 0.86 | (0.67, | 1.1) |
| G/A | E., B. | 120/270 | 72/148 | | 1.1 | (0.74, | 1.6) | | 1.7 | (0.83, | 3.5) |
| rs12302580 | C., B. | 330/843 | 72/195 | | 0.94 | (0.70, | 1.3) | | 0.98 | (0.72, | 1.3) |
| G/C | E., B. | 184/364 | 40/76 | | 1.0 | (0.67, | 1.6) | | 1.5 | (0.64, | 3.7) |
| rs12306963 C/A | E., B. | 164/366 | 48/90 | | 1.2 | (0.75, | 1.9) | | 1.1 | (0.42, | 2.6) |
| rs12314197 | C., B. | 335/866 | 75/246 | | 0.79 | (0.59, | 1.1) | | 0.81 | (0.60, | 1.1) |
| A/G | E., B. | 174/380 | 42/92 | | 1.0 | (0.65, | 1.5) | | 1.0 | (0.65, | 1.5) |
| rs12321826 | C., W. | 294/1075 | 18/73 | | 0.90 | (0.53, | 1.5) | | 0.92 | (0.51, | 1.7) |
| C/T | E., B. | 183/419 | 31/39* | | 1.8 | (1.1, | 3.2)** | | 1.8 | (1.0, | 3.2)** |
|  | E., W. | 434/1626 | 32/138 | | 0.87 | (0.58, | 1.3) | | 0.73 | (0.44, | 1.2) |
| rs12717991 | C., B. | 285/727 | 117/331 | | 0.90 | (0.70, | 1.2) | | 0.93 | (0.72, | 1.2) |
| C/T | E., B. | 159/322 | 57/134 | | 0.86 | (0.58, | 1.3) | | 0.86 | (0.58, | 1.3) |
| rs12721364 | C., B. | 363/975 | 43/137 | | 0.84 | (0.59, | 1.2) | | 0.91 | (0.62, | 1.3) |
| G/A | C., W. | 237/916 | 59/240 | | 0.95 | (0.69, | 1.3) | | 0.91 | (0.64, | 1.3) |
|  | E., B. | 199/394 | 19/68* | | 0.55 | (0.33, | 0.94)** | | 0.31 | (0.12, | 0.81)** |
| rs12721375 | C., W. | 259/1021 | 51/155 | | 1.3 | (0.92, | 1.8) | | 1.4 | (0.96, | 2.1) |
| G/A | E., W. | 412/1556 | 62/208 | | 1.1 | (0.83, | 1.5) | | 0.97 | (0.63, | 1.5) |
| rs12721396 | C., B. | 324/878 | 62/176 | | 0.95 | (0.70, | 1.3) | | 1.0 | (0.74, | 1.4) |
| C/T | C., W. | 201/767 | 71/309 | | 0.88 | (0.65, | 1.2) | | 0.85 | (0.61, | 1.2) |
|  | E., B. | 185/380 | 23/56 | | 0.84 | (0.47, | 1.5) | | 0.84 | (0.47, | 1.5) |
| rs12721397 | C., B. | 324/841 | 80/249 | | 0.83 | (0.63, | 1.1) | | 0.88 | (0.65, | 1.2) |
| A/G | C., W. | 255/953 | 55/209 | | 0.98 | (0.71, | 1.4) | | 0.98 | (0.68, | 1.4) |
|  | E., B. | 168/354 | 46/108 | | 0.90 | (0.61, | 1.3) | | 0.90 | (0.61, | 1.3) |
|  | E., W. | 368/1383 | 94/377 | | 0.94 | (0.73, | 1.2) | | 0.92 | (0.62, | 1.4) |
| rs12721416 | C., B. | 260/735 | 138/341 | | 1.1 | (0.90, | 1.5) | | 1.0 | (0.80, | 1.3) |
| C/T | C., W. | 262/1052 | 42/144 | | 1.2 | (0.81, | 1.7) | | 1.3 | (0.90, | 2.0) |
|  | E., B. | 143/330 | 73/130 | | 1.3 | (0.88, | 1.9) | | 1.3 | (0.88, | 1.9) |
|  | E., W. | 409/1583 | 59/209 | | 1.1 | (0.80, | 1.5) | | 1.1 | (0.73, | 1.8) |
| rs12814512 | C., B. | 241/654 | 157/426 | | 1.0 | (0.79, | 1.3) | | 0.94 | (0.73, | 1.2) |
| G/A | C., W. | 275/1088 | 33/106 | | 1.2 | (0.82, | 1.9) | | 1.6 | (1.0, | 2.4)** |
|  | E., B. | 120/278 | 94/172 | | 1.3 | (0.90, | 1.8) | | 2.2 | (0.81, | 6.2) |
|  | E., W. | 455/1640 | 23/123* | | 0.63 | (0.40, | 0.99)** | | 0.68 | (0.39, | 1.2) |
| rs12814620 | E., B. | 88/259 | 92/173* | | 1.6 | (1.0, | 2.4)** | | 3.0 | (1.1, | 8.0)** |
| T/G | E., W. | 423/1479 | 53/269* | | 0.69 | (0.50, | 0.94)** | | 0.60 | (0.40, | 0.90)** |
| rs12820931 | C., B. | 321/868 | 59/158 | | 1.0 | (0.73, | 1.4) | | 1.0 | (0.72, | 1.4) |
| C/T | C., W. | 274/1066 | 26/98 | | 1.0 | (0.66, | 1.6) | | 1.0 | (0.62, | 1.6) |
|  | E., B. | 178/399 | 30/61 | | 1.1 | (0.71, | 1.7) | | 1.1 | (0.71, | 1.7) |
|  | E., W. | 440/1606 | 34/128 | | 0.97 | (0.65, | 1.4) | | 0.71 | (0.39, | 1.3) |
| rs12831006 | C., B. | 336/982 | 66/132* | | 1.5 | (1.1, | 2.0)** | | 1.4 | (0.96, | 1.9) |
| A/T | C., W. | 241/958 | 71/194* | | 1.5 | (1.1, | 2.0)** | | 1.8 | (1.3, | 2.6)**** |
|  | E., B. | 198/425 | 28/53 | | 1.1 | (0.67, | 1.9) | | 1.1 | (0.67, | 1.9) |
|  | E., W. | 372/1457 | 100/291* | | 1.4 | (1.0, | 1.7)** | | 1.6 | (1.1, | 2.4)** |
| rs1540339 | C., B. | 319/837 | 73/235 | | 0.82 | (0.61, | 1.1) | | 0.87 | (0.64, | 1.2) |
| C/T | E., B. | 173/366 | 45/98 | | 0.97 | (0.63, | 1.5) | | 0.97 | (0.63, | 1.5) |
| rs1544410 | C., B. | 275/734 | 129/324 | | 1.1 | (0.83, | 1.4) | | 1.0 | (0.80, | 1.3) |
| C/T | C., W. | 175/693 | 109/469 | | 0.92 | (0.71, | 1.2) | | 0.97 | (0.72, | 1.3) |
|  | E., B. | 156/303 | 60/147 | | 0.79 | (0.54, | 1.2) | | 0.79 | (0.54, | 1.2) |
|  | E., W. | 264/1013 | 166/649 | | 0.98 | (0.79, | 1.2) | | 1.1 | (0.75, | 1.5) |
| rs17882106 | C., W. | 276/1073 | 40/119 | | 1.3 | (0.89, | 1.9) | | 1.6 | (1.0, | 2.4)** |
| G/A | E., W. | 427/1642 | 57/168 | | 1.3 | (0.95, | 1.8) | | 1.3 | (0.81, | 2.2) |
| rs1989969 | C., W. | 182/714 | 108/416 | | 1.0 | (0.78, | 1.3) | | 0.92 | (0.68, | 1.2) |
| G/A | E., B. | 124/256 | 88/202 | | 0.90 | (0.63, | 1.3) | | 1.6 | (0.75, | 3.3) |
|  | E., W. | 286/1031 | 168/679 | | 0.89 | (0.72, | 1.1) | | 1.1 | (0.80, | 1.6) |
| rs2107301 G/A | E., B. | 169/347 | 51/111 | | 0.94 | (0.61, | 1.5) | | 0.48 | (0.18, | 1.3) |
| rs2189480 | C., B. | 264/665 | 122/391 | | 0.79 | (0.61, | 1.0) | | 0.82 | (0.63, | 1.1) |
| G/T | E., B. | 137/278 | 63/154 | | 0.83 | (0.56, | 1.2) | | 0.83 | (0.56, | 1.2) |
| rs2228570 G/A | E., B. | 158/343 | 42/121 | | 0.75 | (0.47, | 1.2) | | 0.63 | (0.24, | 1.7) |
| rs2238135 | C., B. | 277/777 | 133/321 | | 1.2 | (0.91, | 1.5) | | 1.1 | (0.87, | 1.5) |
| C/G | C., W. | 235/891 | 69/291 | | 0.90 | (0.67, | 1.2) | | 0.93 | (0.67, | 1.3) |
|  | E., B. | 150/344 | 68/134 | | 1.2 | (0.81, | 1.7) | | 1.2 | (0.81, | 1.7) |
|  | E., W. | 360/1354 | 114/408 | | 1.1 | (0.83, | 1.3) | | 0.74 | (0.52, | 1.1) |
| rs2238136 | C., B. | 376/1017 | 30/83 | | 0.98 | (0.63, | 1.5) | | 1.1 | (0.67, | 1.7) |
| C/T | E., B. | 197/438 | 21/34 | | 1.4 | (0.74, | 2.5) | | 1.4 | (0.74, | 2.5) |
|  | E., W. | 340/1276 | 116/388 | | 1.1 | (0.88 | 1.4) | | 0.75 | (0.52, | 1.1) |
| rs2238137 | C., W. | 287/1101 | 19/107 | | 0.68 | (0.41, | 1.1) | | 0.59 | (0.34, | 1.0)** |
| C/T | E., W. | 450/1658 | 34/144 | | 0.87 | (0.59, | 1.3) | | 0.90 | (0.47, | 1.7) |
| rs2238139 | C., B. | 294/790 | 102/280 | | 0.98 | (0.75, | 1.3) | | 0.99 | (0.75, | 1.3) |
| A/G | E., B. | 158/326 | 54/118 | | 0.94 | (0.62, | 1.4) | | 0.94 | (0.62, | 1.4) |
| rs2238140 | C., B. | 217/532 | 185/500 | | 0.91 | (0.72, | 1.1) | | 0.87 | (0.68, | 1.1) |
| A/G | E., B. | 101/229 | 99/225 | | 1.0 | (0.69, | 1.4) | | 1.0 | (0.69, | 1.4) |
| rs2239180 | C., B. | 344/923 | 56/149 | | 1.0 | (0.72, | 1.4) | | 1.0 | (0.73, | 1.5) |
| C/G | C., W. | 266/1047 | 38/147 | | 1.0 | (0.69, | 1.5) | | 1.0 | (0.67, | 1.5) |
|  | E., B. | 195/407 | 31/67 | | 0.97 | (0.59, | 1.6) | | 0.97 | (0.59, | 1.6) |
| rs2239182 C/T | E., B. | 134/262 | 70/170 | | 0.81 | (0.54, | 1.2) | | 0.81 | (0.54, | 1.2) |
| rs2239184 | C., B. | 264/676 | 144/366 | | 1.0 | (0.79, | 1.3) | | 0.99 | (0.77, | 1.3) |
| A/G | C., W. | 156/638 | 142/514 | | 1.1 | (0.88, | 1.5) | | 1.1 | (0.81, | 1.4) |
|  | E., B. | 131/297 | 85/175 | | 1.1 | (0.77, | 1.6) | | 1.1 | (0.77, | 1.6) |
| rs2239186 | C., B. | 394/1065 | 18/61 | | 0.80 | (0.47, | 1.4) | | 0.84 | (0.47, | 1.5) |
| A/G | E., B. | 218/440 | 10/40 | | 0.50 | (0.23, | 1.1) | | 0.50 | (0.23, | 1.1) |
| rs2240105 A/G | E., B. | 158/364 | 42/74 | | 1.3 | (0.82, | 2.1) | | 1.3 | (0.82, | 2.1) |
| rs2246001 | C., B. | 280/714 | 112/358 | | 0.80 | (0.62, | 1.0) | | 0.79 | (0.61, | 1.0) |
| G/A | C., W. | 246/984 | 60/184 | | 1.3 | (0.94, | 1.8) | | 1.3 | (0.92, | 1.9) |
|  | E., B. | 156/303 | 60/151 | | 0.77 | (0.52, | 1.2) | | 0.77 | (0.33, | 1.8) |
|  | E., W. | 392/1520 | 151/270 | | 1.0 | (0.78, | 1.4) | | 1.2 | (0.82, | 1.9) |
| rs2254210  G/A | E., B. | 143/286 | 53/72 | | 0.80 | (0.52, | 1.2) | | 1.0 | (0.47, | 2.1) |
| rs2283342 | C., W. | 269/1016 | 41/184 | | 0.84 | (0.58, | 1.2) | | 0.83 | (0.55, | 1.2) |
| A/G | E., B. | 202/460 | 12/32 | | 0.85 | (0.41, | 1.8) | | 0.85 | (0.41, | 1.8) |
|  | E., W. | 406/1517 | 72/257 | | 1.1 | (0.79, | 1.4) | | 1.0 | (0.68, | 1.6) |
| rs2408875 G/T | E., B. | 118/267 | 82/153 | | 1.2 | (0.84, | 1.8) | | 1.2 | (0.84, | 1.8) |
| rs2408876 | C., B. | 208/552 | 192/516 | | 0.99 | (0.78, | 1.2) | | 1.0 | (0.79, | 1.3) |
| T/C | C., W. | 160/654 | 144/480 | | 1.2 | (0.95, | 1.6) | | 1.2 | (0.89, | 1.6) |
|  | E., B. | 107/245 | 103/219 | | 1.1 | (0.74, | 1.6) | | 0.71 | (0.28, | 1.8) |
|  | E., W. | 250/1020 | 174/724 | | 0.98 | (0.79, | 1.2) | | 0.93 | (0.68, | 1.3) |
| rs2408877 | C., B. | 378/1033 | 32/77 | | 1.1 | (0.74, | 1.7) | | 1.3 | (0.81, | 2.0) |
| A/T | C., W. | 248/932 | 60/226 | | 1.0 | (0.73, | 1.4) | | 1.1 | (0.81, | 1.6) |
|  | E., B. | 202/430 | 14/46 | | 0.65 | (0.33, | 1.3) | | 0.65 | (0.33, | 1.3) |
|  | E., W. | 376/1465 | 86/289 | | 1.2 | (0.89, | 1.5) | | 0.90 | (0.60, | 1.3) |
| rs2525045 | C., B. | 314/872 | 96/218 | | 1.2 | (0.93, | 1.6) | | 1.2 | (0.88, | 1.6) |
| G/A | C., W. | 294/1110 | 18/100 | | 0.68 | (0.40, | 1.1) | | 0.58 | (0.33, | 1.0) |
|  | E., B. | 164/367 | 46/95 | | 1.1 | (0.71, | 1.7) | | 1.4 | (0.71, | 2.6) |
|  | E., W. | 443/1655 | 47/157 | | 1.1 | (0.79, | 1.6) | | 1.5 | (0.96, | 2.5) |
| rs2525046 | C., W. | 163/700 | 125/414 | | 1.3 | (1.0, | 1.7) | | 1.3 | (0.95, | 1.7) |
| C/T | E., B. | 155/331 | 53/97 | | 1.2 | (0.76, | 1.8) | | 1.2 | (0.76, | 1.8) |
|  | E., W. | 264/992 | 172/622 | | 1.0 | (0.84, | 1.3) | | 1.0 | (0.73, | 1.4) |
| rs2525051 | C., B. | 179/454 | 187/534 | | 0.89 | (0.70, | 1.1) | | 0.84 | (0.65, | 1.1) |
| G/C | C., W. | 170/587 | 114/505 | | 0.78 | (0.60, | 1.0) | | 0.79 | (0.59, | 1.1) |
|  | E., B. | 85/197 | 115/263 | | 1.0 | (0.72, | 1.4) | | 1.1 | (0.46, | 2.6) |
| rs2525053 | C., B. | 188/523 | 194/527 | | 1.0 | (0.81, | 1.3) | | 0.96 | (0.75, | 1.2) |
| T/C | C., W. | 202/739 | 100/395 | | 0.93 | (0.71, | 1.2) | | 0.95 | (0.70, | 1.3) |
|  | E., B. | 93/222 | 111/240 | | 1.1 | (0.78, | 1.6) | | 1.7 | (0.91, | 3.3) |
|  | E., W. | 327/1115 | 127/553* | | 0.78 | (0.62, | 0.98)** | | 0.74 | (0.53, | 1.0) |
| rs2544027 | C., B. | 246/628 | 146/420 | | 0.89 | (0.70, | 1.1) | | 0.89 | (0.70, | 1.1) |
| G/A | C., W. | 150/598 | 142/558 | | 1.0 | (0.78, | 1.3) | | 1.0 | (0.76, | 1.3) |
|  | E., B. | 138/286 | 74/180 | | 0.85 | (0.59, | 1.2) | | 1.4 | (0.61, | 3.2) |
|  | E., W. | 218/866 | 232/834 | | 1.1 | (0.90, | 1.4) | | 1.3 | (0.94, | 1.8) |
| rs2544028 | C., W. | 163/703 | 115/433 | | 1.2 | (0.88, | 1.5) | | 1.2 | (0.87, | 1.6) |
| A/T | E., B. | 152/328 | 52/124 | | 0.90 | (0.60, | 1.4) | | 0.90 | (0.60, | 1.4) |
| rs2544030 | C., B. | 294/838 | 110/258 | | 1.2 | (0.94, | 1.6) | | 1.2 | (0.93, | 1.6) |
| G/A | E., B. | 171/357 | 47/105 | | 0.93 | (0.61, | 1.4) | | 0.98 | (0.37, | 2.6) |
|  | E., W. | 336/1267 | 128/501 | | 0.96 | (0.77, | 1.2) | | 1.1 | (0.82, | 1.6) |
| rs2544036 C/G | E., B. | 136/29 | 74/156 | | 1.0 | (0.68, | 1.5) | | 1.0 | (0.68, | 1.5) |
| rs2544037 | C., B. | 308/801 | 100/283 | | 0.92 | (0.71, | 1.2) | | 0.93 | (0.70, | 1.2) |
| A/G | C., W. | 174/701 | 128/463 | | 1.1 | (0.86, | 1.4) | | 1.2 | (0.87, | 1.5) |
|  | E., B. | 163/344 | 41/116 | | 0.75 | (0.48, | 1.2) | | 0.75 | (0.48, | 1.2) |
| rs2544039 | C., W. | 246/1040 | 56/158* | | 1.5 | (1.1, | 2.1)** | | 1.5 | (1.0, | 2.1)** |
| C/T | E., B. | 187/397 | 33/73 | | 0.96 | (0.57, | 1.6) | | 0.96 | (0.57, | 1.6) |
| rs2544042 G/A | E., B. | 181/375 | 29/81 | | 0.74 | (0.46, | 1.2) | | 0.73 | (0.22, | 2.4) |
| rs2853559 G/A | E., B. | 186/369 | 30/89 | | 0.67 | (0.40, | 1.1) | | 0.86 | (0.25, | 2.9) |
| rs2853561 | C., W. | 113/424 | 183/714 | | 0.96 | (0.74, | 1.3) | | 0.83 | (0.62, | 1.1) |
| C/T | E., B. | 139/298 | 69/142 | | 1.0 | (0.71, | 1.5) | | 1.0 | (0.71, | 1.5) |
| rs2853563 C/T | E., B. | 182/411 | 18/41 | | 0.99 | (0.50, | 2.0) | | 0.99 | (0.50, | 2.0) |
| rs2853564 | C., W. | 194/759 | 110/395 | | 1.1 | (0.84, | 1.4) | | 0.93 | (0.69, | 1.3) |
| A/G | E., B. | 196/420 | 20/64 | | 0.67 | (0.39, | 1.2) | | 0.67 | (0.39, | 1.1) |
|  | E., W. | 296/1061 | 158/679 | | 0.83 | (0.67, | 1.0) | | 1.1 | (0.78, | 1.6) |
| rs34379708 | C., W. | 279/1028 | 31/118 | | 0.97 | (0.64, | 1.5) | | 1.1 | (0.70, | 1.7) |
| G/A | E., B. | 156/352 | 46/98 | | 1.1 | (0.68, | 1.7) | | 0.77 | (0.28, | 2.2) |
| rs34922454  A/C | E., B. | 107/251 | 83/177 | | 1.1 | (0.74, | 1.6) | | 1.1 | (0.74, | 1.6) |
| rs35609792 A/G | C., B. | 319/845 | 77/235 | | 0.87 | (0.65, | 1.2) | | 0.88 | (0.64, | 1.2) |
| rs36029544 | C., W. | 281/1047 | 35/141 | | 0.92 | (0.62, | 1.4) | | 0.98 | (0.64, | 1.5) |
| A/G | E., W. | 431/1627 | 45/205 | | 0.83 | (0.59, | 1.2) | | 0.52 | (0.31, | 0.86)** |
| rs3782905 | C., W. | 190/728 | 94/390 | | 0.92 | (0.70, | 1.2) | | 0.99 | (0.73, | 1.3) |
| G/C | E., B. | 163/340 | 37/116 | | 0.67 | (0.42, | 1.1) | | 0.66 | (0.25, | 1.7) |
| rs3815129 | C., B. | 312/834 | 102/278 | | 0.98 | (0.76, | 1.3) | | 0.98 | (0.75, | 1.3) |
| T/C | C., W. | 254/1027 | 46/177 | | 1.1 | (0.74, | 1.5) | | 1.1 | (0.71, | 1.6) |
|  | E., B. | 149/344 | 53/120 | | 1.0 | (0.68, | 1.5) | | 0.96 | (0.42, | 2.2) |
| rs3819545 | C., B. | 313/796 | 105/270 | | 0.99 | (0.76, | 1.3) | | 1.0 | (0.79, | 1.4) |
| A/G | E., B. | 172/345 | 52/117 | | 0.89 | (0.59, | 1.4) | | 0.89 | (0.59, | 1.3) |
|  | E., W. | 279/1092 | 179/668 | | 1.1 | (0.85, | 1.3) | | 0.76 | (0.54, | 1.1) |
| rs3847987 C/A | E., B. | 197/434 | 25/30* | | 1.8 | (1.0, | 3.3)** | | 1.0 | (0.41, | 2.6) |
| rs3922882 | C., B. | 336/893 | 80/205 | | 1.0 | (0.78, | 1.4) | | 1.1 | (0.81, | 1.5) |
| C/G | C., W. | 210/758 | 98/390 | | 0.91 | (0.69, | 1.2) | | 0.80 | (0.59, | 1.1) |
|  | E., B. | 186/396 | 32/74 | | 0.92 | (0.58, | 1.5) | | 0.92 | (0.58, | 1.5) |
|  | E., W. | 294/1085 | 156/639 | | 0.90 | (0.72, | 1.1) | | 1.2 | (0.85, | 1.7) |
| rs4073729 | C., B. | 294/791 | 112/297 | | 1.0 | (0.79, | 1.3) | | 1.0 | (0.77, | 1.3) |
| G/A | C., W. | 260/994 | 60/188 | | 1.2 | (0.88, | 1.7) | | 1.6 | (1.1, | 2.2)** |
|  | E., B. | 170/338 | 54/132 | | 0.81 | (0.55, | 1.2) | | 0.64 | (0.33, | 1.2) |
| rs4237855 A/G | E., B. | 164/318 | 54/128 | | 0.82 | (0.54, | 1.3) | | 0.82 | (0.54, | 1.2) |
| rs4307775 | C., W. | 228/875 | 68/261 | | 1.0 | (0.74, | 1.4) | | 0.95 | (0.68, | 1.3) |
| C/G | E., B. | 211/443 | 13/33 | | 0.83 | (0.40, | 1.7) | | 0.83 | (0.40, | 1.7) |
|  | E., W. | 351/1287 | 103/393 | | 0.96 | (0.75, | 1.2) | | 0.74 | (0.51, | 1.1) |
| rs4334089 A/G | E., B. | 127/250 | 75/194 | | 0.76 | (0.52, | 1.1) | | 0.76 | (0.52, | 1.1) |
| rs4340112 C/T | C., B. | 331/851 | 47/121 | | 1.0 | (0.70, | 1.4) | | 1.0 | (0.70, | 1.5) |
| rs4393380 | C., W. | 236/915 | 56/211 | | 1.0 | (0.74, | 1.4) | | 1.3 | (0.91, | 1.9) |
| G/T | E., W. | 364/1419 | 86/255* | | 1.3 | (1.0, | 1.7)** | | 1.3 | (0.86, | 2.1) |
| rs4506714 | C., W. | 203/814 | 85/354 | | 0.96 | (0.73, | 1.3) | | 0.90 | (0.67, | 1.2) |
| C/T | E., B. | 204/445 | 14/37 | | 0.83 | (0.42, | 1.6) | | 0.83 | (0.42, | 1.6) |
|  | E., W. | 339/1172 | 129/498 | | 0.90 | (0.71, | 1.1) | | 1.2 | (0.82, | 1.7) |
| rs4576885 G/A | E., B. | 171/392 | 27/58 | | 1.1 | (0.63, | 1.8) | | 2.0 | (0.86, | 4.7) |
| rs4583039 A/G | E., B. | 94/219 | 86/209 | | 0.96 | (0.65, | 1.4) | | 0.96 | (0.65, | 1.4) |
| rs4760624 G/T | C., B. | 364/1022 | 28/52 | | 1.5 | (0.94, | 2.4) | | 1.5 | (0.91, | 2.6) |
| rs4760650 | C., B. | 342/880 | 64/198 | | 0.83 | (0.61, | 1.1) | | 0.86 | (0.63, | 1.2) |
| G/T | C., W. | 204/851 | 62/253 | | 1.0 | (0.74, | 1.4) | | 0.92 | (0.64, | 1.3) |
|  | E., B. | 173/392 | 35/70 | | 1.1 | (0.74, | 1.7) | | 1.1 | (0.74, | 1.7) |
|  | E., W. | 351/1265 | 85/377 | | 0.81 | (0.62, | 1.1) | | 0.91 | (0.62, | 1.4) |
| rs4760671 | C., B. | 384/1008 | 40/126 | | 0.83 | (0.57, | 1.2) | | 0.93 | (0.61, | 1.4) |
| C/G | C., W. | 179/666 | 125/468 | | 0.99 | (0.77, | 1.3) | | 0.89 | (0.68, | 1.2) |
|  | E., B. | 206/426 | 22/54 | | 0.84 | (0.48, | 1.5) | | 0.84 | (0.48, | 1.5) |
|  | E., W. | 278/944 | 190/794* | | 0.81 | (0.66, | 1.0)** | | 1.0 | (0.75, | 1.4) |
| rs55713656 | C., W. | 238/950 | 56/202 | | 1.1 | (0.80, | 1.5) | | 1.4 | (0.95, | 1.9) |
| G/A | E., B. | 183/390 | 25/54 | | 0.99 | (0.56, | 1.7) | | 0.60 | (0.19, | 1.9) |
| rs55900360 | C., B. | 371/1011 | 33/117 | | 0.77 | (0.51, | 1.2) | | 0.73 | (0.48, | 1.1) |
| A/G | C., W. | 276/1041 | 22/127 | | 0.65 | (0.41, | 1.1) | | 0.57 | (0.33, | 0.99)** |
|  | E., B. | 200/438 | 18/44 | | 0.90 | (0.49, | 1.7) | | 0.90 | (0.49, | 1.7) |
| rs58789572 C/T | E., B. | 175/398 | 39/68 | | 1.3 | (0.79, | 2.2) | | 1.3 | (0.79, | 2.2) |
| rs60025549 | C., W. | 293/1116 | 17/78 | | 0.83 | (0.48, | 1.4) | | 0.71 | (0.38, | 1.3) |
| T/A | E., B. | 190/376 | 28/92* | | 0.60 | (0.38, | 0.96)** | | 0.86 | (0.38, | 2.0) |
| rs61917655 | C., W. | 291/1076 | 23/114 | | 0.75 | (0.47, | 1.2) | | 0.59 | (0.34, | 1.0) |
| C/T | E., W. | 416/1623 | 50/185 | | 1.1 | (0.76, | 1.5) | | 0.97 | (0.56, | 1.7) |
| rs6580638 G/A | E., B. | 160/378 | 54/70** | | 1.8 | (1.2, | 2.8)*** | | 3.9 | (1.4, | 11.5)** |
| rs6580642 | C., B. | 377/1039 | 49/111 | | 1.2 | (0.85, | 1.7) | | 1.3 | (0.89, | 1.9) |
| C/T | C., W. | 253/1012 | 55/178 | | 1.2 | (0.89, | 1.7) | | 1.2 | (0.82, | 1.7) |
|  | E., B. | 196/434 | 26/50 | | 1.2 | (0.69, | 1.9) | | 1.2 | (0.69, | 1.9) |
|  | E., W. | 399/1546 | 73/258 | | 1.1 | (0.83, | 1.5) | | 1.5 | (1.0, | 2.2)** |
| rs7132324 | C., B. | 401/1061 | 21/73 | | 0.76 | (0.46, | 1.3) | | 0.81 | (0.48, | 1.4) |
| C/T | E., B. | 200/434 | 16/42 | | 0.83 | (0.41, | 1.7) | | 0.83 | (0.41, | 1.6) |
| rs7139166 | C., B. | 383/1026 | 39/110 | | 0.95 | (0.65, | 1.4) | | 1.0 | (0.67, | 1.6) |
| C/G | C., W. | 190/664 | 112/470 | | 0.83 | (0.64, | 1.1) | | 0.76 | (0.57, | 1.0) |
|  | E., B. | 205/429 | 15/53 | | 0.59 | (0.33, | 1.1) | | 0.59 | (0.33, | 1.1) |
|  | E., W. | 266/968 | 176/734 | | 0.87 | (0.70, | 1.1) | | 0.93 | (0.66, | 1.3) |
| rs7300088 | C., B. | 121/309 | 283/785 | | 0.92 | (0.72, | 1.2) | | 0.88 | (0.67, | 1.2) |
| A/G | C., W. | 221/924 | 85/252* | | 1.4 | (1.1, | 1.9)** | | 1.8 | (1.3, | 2.4)**** |
|  | E., B. | 65/157 | 145/307 | | 1.1 | (0.78, | 1.7) | | 2.4 | (1.1, | 5.5)** |
|  | E., W. | 353/1444 | 115/346* | | 1.4 | (1.1, | 1.7) | | 1.4 | (1.0, | 2.0) |
| rs7305032 | C., W. | 156/659 | 124/463 | | 1.1 | (0.87, | 1.5) | | 1.1 | (0.84, | 1.5) |
| A/G | E., B. | 136/332 | 58/130 | | 1.1 | (0.73, | 1.6) | | 1.1 | (0.73, | 1.6) |
| rs7308216 | C., B. | 162/451 | 244/643 | | 1.1 | (0.84, | 1.3) | | 1.0 | (0.78, | 1.3) |
| G/C | C., W. | 227/932 | 77/260 | | 1.2 | (0.91, | 1.6) | | 1.4 | (1.0, | 2.0)** |
|  | E., B. | 91/222 | 133/254 | | 1.3 | (0.90, | 1.8) | | 1.8 | (0.86, | 3.8) |
|  | E., W. | 359/1438 | 111/350 | | 1.3 | (1.0, | 1.6) | | 1.4 | (0.95, | 2.0) |
| rs73109883 | C., B. | 366/974 | 62/162 | | 1.0 | (0.74, | 1.4) | | 0.97 | (0.71, | 1.3) |
| G/A | C., W. | 250/929 | 66/233 | | 1.1 | (0.77, | 1.4) | | 1.1 | (0.82, | 1.6) |
|  | E., B. | 196/405 | 24/65 | | 0.76 | (0.43, | 1.4) | | 1.6 | (0.59, | 4.4) |
| rs731236 A/G | E., B. | 155/310 | 53/110 | | 0.96 | (0.62, | 1.5) | | 0.96 | (0.62, | 1.5) |
| rs7316233 | C., B. | 361/974 | 43/122 | | 0.95 | (0.66, | 1.4) | | 0.95 | (0.65, | 1.4) |
| A/G | C., W. | 285/1054 | 31/134 | | 0.86 | (0.57, | 1.3) | | 0.83 | (0.52, | 1.3) |
|  | E., B. | 187/396 | 33/70 | | 1.0 | (0.62, | 1.6) | | 1.0 | (0.62, | 1.6) |
| rs73291248 T/C | C., B. | 333/897 | 33/109 | | 0.82 | (0.54, | 1.2) | | 0.69 | (0.45, | 1.1) |
| rs73293224 | C., B. | 361/1009 | 35/101 | | 0.97 | (0.65, | 1.5) | | 1.0 | (0.67, | 1.6) |
| C/T | E., B. | 199/414 | 17/46 | | 0.77 | (0.42, | 1.4) | | 0.77 | (0.42, | 1.4) |
| rs73295192 C/A | E., B. | 192/378 | 28/84 | | 0.66 | (0.39, | 1.1) | | 0.52 | (0.21, | 1.3) |
| rs739837 T/G | E., B. | 107/247 | 95/183 | | 1.2 | (0.82, | 1.7) | | 1.7 | (0.79, | 3.6) |
| rs739842 | C., B. | 371/1015 | 43/97 | | 1.2 | (0.83, | 1.8) | | 1.4 | (0.92, | 2.0) |
| C/T | E., B. | 189/410 | 25/52 | | 1.0 | (0.58, | 1.9) | | 1.0 | (0.58, | 1.9) |
| rs74088121 | C., B. | 350/922 | 48/138 | | 0.92 | (0.65, | 1.3) | | 0.91 | (0.63, | 1.3) |
| G/A | E., B. | 190/386 | 16/66* | | 0.49 | (0.25, | 0.97)** | | 0.22 | (0.07, | 0.73)** |
| rs74356534 | C., B. | 377/1017 | 41/129 | | 0.86 | (0.59, | 1.2) | | 0.83 | (0.56, | 1.2) |
| C/T | E., B. | 198/431 | 18/47 | | 0.83 | (0.47, | 1.5) | | 0.83 | (0.47, | 1.5) |
| rs757344 | C., B. | 188/523 | 206/541 | | 1.1 | (0.84, | 1.4) | | 1.0 | (0.79, | 1.3) |
| C/T | C., W. | 135/488 | 155/624 | | 0.90 | (0.69, | 1.2) | | 0.97 | (0.73, | 1.3) |
|  | E., B. | 94/237 | 114/213 | | 1.4 | (0.96, | 1.9) | | 1.3 | (0.96, | 1.9) |
| rs77766878 | C., B. | 336/863 | 78/251 | | 0.80 | (0.60, | 1.1) | | 0.76 | (0.56, | 1.0) |
| C/G | E., B. | 187/404 | 33/84 | | 0.85 | (0.54, | 1.3) | | 0.85 | (0.54, | 1.3) |
| rs7956152 | C., B. | 355/943 | 43/129 | | 0.89 | (0.61, | 1.3) | | 0.78 | (0.53, | 1.2) |
| A/T | E., B. | 188/431 | 22/45 | | 1.1 | (0.63, | 2.0) | | 1.1 | (0.63, | 2.0) |
| rs7958910 | C., B. | 251/720 | 123/336 | | 1.1 | (0.82, | 1.4) | | 1.0 | (0.77, | 1.3) |
| A/T | C., W. | 206/744 | 88/378 | | 0.84 | (0.64, | 1.1) | | 0.84 | (0.62, | 1.1) |
|  | E., B. | 138/302 | 62/134 | | 1.0 | (0.66, | 1.5) | | 1.0 | (0.66, | 1.5) |
| rs7959280 | C., B. | 186/516 | 188/492 | | 1.1 | (0.84, | 1.3) | | 1.0 | (0.80, | 1.3) |
| A/G | C., W. | 207/749 | 77/339 | | 0.82 | (0.61, | 1.1) | | 0.82 | (0.59, | 1.1) |
|  | E., B. | 111/241 | 95/203 | | 1.0 | (0.70, | 1.5) | | 1.0 | (0.70, | 1.5) |
| rs7959281 | C., B. | 295/767 | 99/303 | | 0.85 | (0.65, | 1.1) | | 0.87 | (0.66, | 1.1) |
| C/G | C., W. | 252/970 | 48/210 | | 0.88 | (0.62, | 1.2) | | 0.81 | (0.56, | 1.2) |
|  | E., B. | 164/339 | 46/117 | | 0.81 | (0.53, | 1.3) | | 0.81 | (0.53, | 1.3) |
| rs7962898 | C., B. | 257/670 | 157/398 | | 1.0 | (0.81, | 1.3) | | 1.1 | (0.83, | 1.4) |
| C/T | C., W. | 152/517 | 140/627* | | 0.76 | (0.59, | 0.98)** | | 0.79 | (0.60, | 1.1) |
|  | E., B. | 145/287 | 59/155 | | 0.75 | (0.52, | 1.1) | | 0.75 | (0.52, | 1.1) |
| rs7963776 A/G | E., B. | 123/270 | 81/156 | | 1.1 | (0.77, | 1.7) | | 1.1 | (0.77, | 1.7) |
| rs7965281 | C., B. | 237/667 | 153/399 | | 1.1 | (0.85, | 1.4) | | 1.0 | (0.82, | 1.4) |
| A/G | C., W. | 143/560 | 131/580 | | 0.88 | (0.68, | 1.2) | | 0.95 | (0.71, | 1.3) |
|  | E., B. | 136/298 | 70/174 | | 0.88 | (0.61, | 1.3) | | 0.88 | (0.61, | 1.3) |
| rs7965397 | C., B. | 370/1015 | 46/123 | | 0.97 | (0.68, | 1.4) | | 0.95 | (0.65, | 1.4) |
| T/G | C., W. | 196/730 | 104/422 | | 0.92 | (0.70, | 1.2) | | 0.91 | (0.68, | 1.2) |
|  | E., B. | 196/401 | 30/63 | | 0.97 | (0.58, | 1.7) | | 0.66 | (0.25, | 1.7) |
|  | E., W. | 287/1064 | 145/636 | | 0.85 | (0.68, | 1.1) | | 0.72 | (0.51, | 1.0) |
| rs7965943 | C., W. | 186/725 | 102/419 | | 0.95 | (0.72, | 1.2) | | 0.81 | (0.60, | 1.1) |
| T/G | E., B. | 83/179 | 131/275 | | 1.0 | (0.72, | 1.5) | | 0.64 | (0.31, | 1.3) |
|  | E., W. | 269/996 | 167/676 | | 0.91 | (0.74, | 1.1) | | 1.1 | (0.79, | 1.6) |
| rs7966244 | C., B. | 373/1044 | 29/68 | | 1.2 | (0.76, | 1.9) | | 1.1 | (0.70, | 1.8) |
| A/T | E., B. | 204/442 | 20/26 | | 1.7 | (0.87, | 3.2) | | 1.7 | (0.87, | 3.2) |
| rs7968585 | C., B. | 162/417 | 238/651 | | 0.94 | (0.74, | 1.2) | | 0.96 | (0.75, | 1.2) |
| C/T | C., W. | 161/559 | 145/569 | | 0.88 | (0.69, | 1.1) | | 0.98 | (0.74, | 1.3) |
|  | E., B. | 91/190 | 127/264 | | 1.0 | (0.70, | 1.4) | | 1.4 | (0.72, | 2.8) |
|  | E., W. | 224/854 | 232/858 | | 1.0 | (0.84, | 1.3) | | 1.3 | (0.93, | 1.8) |
| rs7971418 A/C | E., B. | 111/244 | 87/178 | | 1.1 | (0.72, | 1.6) | | 1.1 | (0.72, | 1.6) |
| rs7974353 | C., B. | 374/1041 | 40/101 | | 1.1 | (0.75, | 1.6) | | 1.1 | (0.75, | 1.7) |
| C/T | C., W. | 235/1001 | 59/167* | | 1.5 | (1.1, | 2.1)** | | 1.5 | (1.0, | 2.1)** |
|  | E., B. | 202/430 | 20/52 | | 0.82 | (0.48, | 1.4) | | 0.82 | (0.48, | 1.4) |
| rs7974708 | C., B. | 323/871 | 81/233 | | 0.94 | (0.71, | 1.2) | | 0.89 | (0.66, | 1.2) |
| T/C | C., W. | 182/718 | 106/412 | | 1.0 | (0.78, | 1.3) | | 1.1 | (0.81, | 1.5) |
|  | E., B. | 170/356 | 46/108 | | 0.89 | (0.59, | 1.4) | | 1.4 | (0.61, | 3.3) |
| rs7975128 | C., B. | 272/738 | 132/342 | | 1.1 | (0.82, | 1.3) | | 1.1 | (0.82, | 1.4) |
| G/A | C., W. | 170/651 | 122/505 | | 0.93 | (0.71, | 1.2) | | 0.95 | (0.72, | 1.3) |
|  | E., B. | 150/311 | 54/165* | | 0.68 | (0.46, | 1.0) | | 0.87 | (0.39, | 1.9) |
|  | E., W. | 264/1009 | 198/695 | | 1.1 | (0.88, | 1.3) | | 1.3 | (0.93, | 1.8) |
| rs7975232 | C., B. | 320/862 | 64/162 | | 1.1 | (0.78, | 1.5) | | 0.99 | (0.71, | 1.4) |
| A/C | E., B. | 169/400 | 62/62 | | 1.1 | (0.65, | 1.9) | | 1.1 | (0.65, | 1.9) |
| rs7979360 | C., B. | 249/669 | 157/405 | | 1.0 | (0.82, | 1.3) | | 0.98 | (0.77, | 1.3) |
| A/G | E., B. | 139/292 | 75/154 | | 1.0 | (0.71, | 1.5) | | 1.0 | (0.71, | 1.5) |
| rs886441 | C., B. | 243/700 | 129/350 | | 1.1 | (0.83, | 1.4) | | 1.0 | (0.79, | 1.3) |
| A/G | C., W. | 237/955 | 61/191 | | 1.3 | (0.93, | 1.8) | | 1.3 | (0.91, | 1.8) |
|  | E., B. | 119/282 | 81/154 | | 1.3 | (0.87, | 1.8) | | 1.2 | (0.87, | 1.8) |
|  | E., W. | 377/1417 | 77/289 | | 1.0 | (0.76, | 1.3) | | 1.4 | (0.94, | 2.0) |
| rs987849 A/G | E., B. | 151/348 | 45/114 | | 0.91 | (0.58, | 1.4) | | 0.91 | (0.58, | 1.4) |

*p <0.05 based on a chi-square test. **ORs and 95% CI that reach statistical significance at p<0.05. ***ORs and 95% CI that reach statistical significance at p<0.01. ****ORs and 95% CI that reach statistical significance at p<0.001.

CI, confidence interval; C., B., CPP study, Black race; C., W., CPP study, White race; E., B., EVITA study, Black race; E., W., EVITA study, White race; *VDR,* vitamin D receptor

a Groups not shown for each SNP did not pass quality control steps.

b Adjusted for 25(OH)D concentration, maternal age, smoking, body mass index, percent African ancestry (Black mothers only), site (CPP only), year of blood draw (EVITA only), and batch (EVITA only). Significance of the associations are given by symbols.

Supplemental Table 2: Association between minor alleles of *GC* variants and the risk of preeclampsia compared with major alleles by maternal race and study.^a^

| Variant name | Study, Race | Number of controls [minor/major alleles] | Number of cases [minor/major alleles] | Univariate Analysis  Odds Ratio (95%CI) | | | Multivariate Analysis  Odds Ratio (95%CI) ^b^ | | |
| --- | --- | --- | --- | --- | --- | --- | --- | --- | --- |
| rs114138315 | C., W. | 256/988 | 34/142 | 0.92 | (0.62, | 1.4) | 1.0 | (0.66, | 1.5) |
| T/C | E., B. | 162/366 | 32/86 | 0.84 | (0.49, | 1.4) | 1.5 | (0.69, | 3.2) |
|  | E., W. | 400/1479 | 40/193 | 0.77 | (0.54, | 1.1) | 0.82 | (0.48, | 1.4) |
| rs114522037 | C., W. | 297/1094 | 15/82 | 0.67 | (0.38, | 1.2) | 0.70 | (0.38, | 1.3) |
| T/A | E., B. | 172/348 | 24/74 | 0.66 | (0.39, | 1.1) | 1.8 | (0.74, | 4.3) |
|  | E., W. | 407/1606 | 27/104 | 1.0 | (0.66, | 1.6) | 1.3 | (0.65, | 2.6) |
| rs114660378 G/T | E., B. | 200/429 | 10/31 | 0.69 | (0.29, | 1.6) | 0.70 | (0.29, | 1.6) |
| rs11723727 C/G | E., B. | 180/400 | 34/54 | 1.4 | (0.83, | 2.4) | 1.4 | (0.83, | 2.4) |
| rs11732451 | C., W. | 231/947 | 61/207 | 1.2 | (0.88, | 1.7) | 1.1 | (0.75, | 1.5) |
| A/G | E., B. | 199/446 | 13/22 | 1.3 | (0.60, | 2.9) | 1.3 | (0.60, | 2.9) |
| rs12512631 | C., B. | 233/661 | 141/369 | 1.1 | (0.85, | 1.4) | 1.1 | (0.85, | 1.4) |
| T/C | E., B. | 104/296 | 88/132* | 1.9 | (1.3, | 2.8)**** | 3.0 | (1.2, | 7.1)** |
| rs13117483 C/T | E., B. | 200/411 | 20/53 | 0.78 | (0.44, | 1.4) | 0.78 | (0.44, | 1.4) |
| rs13150174 | C., B. | 315/844 | 79/220 | 0.96 | (0.72, | 1.3) | 0.94 | (0.69, | 1.3) |
| A/T | C., W. | 188/773 | 98/365 | 1.1 | (0.84, | 1.5) | 1.0 | (0.77, | 1.4) |
|  | E., W. | 293/1157 | 121/477 | 1.0 | (0.79, | 1.3) | 1.0 | (0.69, | 1.6) |
| rs148488346 | C., B. | 314/817 | 86/201 | 1.1 | (0.84, | 1.5) | 1.1 | (0.82, | 1.5) |
| G/A | C., W. | 269/1020 | 35/132 | 1.0 | (0.68, | 1.5) | 0.92 | (0.59, | 1.4) |
|  | E., B. | 165/317 | 35/89 | 0.76 | (0.48, | 1.2) | 0.40 | (0.15, | 1.0) |
| rs1491714 C/T | C., W. | 285/1077 | 31/115 | 1.0 | (0.67, | 1.5) | 1.1 | (0.66, | 1.7) |
| rs1491719 T/C | C., W. | 254/942 | 36/208* | 0.64 | (0.44, | 0.94)** | 0.62 | (0.40, | 0.97)** |
| rs1609020 A/C | E., B. | 150/296 | 56/104 | 1.1 | (0.68, | 1.7) | 1.1 | (0.68, | 1.7) |
| rs16845007 | C., W. | 288/1108 | 14/96* | 0.56 | (0.32, | 1.0)** | 0.66 | (0.35, | 1.2) |
| T/C | E., B. | 132/333 | 48/91 | 1.3 | (0.83, | 2.1) | 1.2 | (0.39, | 3.5) |
|  | E., W. | 430/1609 | 28/111 | 0.94 | (0.62, | 1.4) | 0.84 | (0.41, | 1.7) |
| rs16846876 | C., B. | 377/944 | 21/94* | 0.56 | (0.34, | 0.91)** | 0.60 | (0.36, | 1.0) |
| A/T | C., W. | 245/960 | 27/134 | 0.79 | (0.51, | 1.2) | 0.91 | (0.56, | 1.5) |
|  | E., B. | 190/384 | 20/44 | 0.92 | (0.51, | 1.6) | 0.92 | (0.51, | 1.6) |
|  | E., W. | 374/1390 | 50/228 | 0.82 | (0.59, | 1.1) | 0.62 | (0.35, | 1.1) |
| rs16846893 | C., W. | 242/986 | 12/54 | 0.91 | (0.48, | 1.7) | 0.97 | (0.50, | 1.9) |
| A/C | E., B. | 181/344 | 17/44 | 0.73 | (0.39, | 1.4) | 0.73 | (0.39, | 1.4) |
|  | E., W. | 388/1412 | 18/88 | 0.74 | (0.44, | 1.3) | 1.1 | (0.50, | 2.4) |
| rs16847015 | C., B. | 330/878 | 46/136 | 0.90 | (0.63, | 1.3) | 0.95 | (0.65, | 1.4) |
| C/A | C., W. | 283/1108 | 21/60 | 1.4 | (0.82, | 2.3) | 1.3 | (0.73, | 2.2) |
|  | E., B. | 163/351 | 31/61 | 1.1 | (0.64, | 1.9) | 1.4 | (0.40, | 4.6) |
|  | E., W. | 422/1587 | 30/117 | 0.96 | (0.64, | 1.5) | 0.83 | (0.49, | 1.4) |
| rs16847019 G/A | E., B. | 191/398 | 15/60* | 0.52 | (0.28, | 0.97)** | 0.31 | (0.12, | 0.79)** |
| rs16847036 A/G | C., W. | 277/1057 | 15/83 | 0.69 | (0.39, | 1.2) | 0.74 | (0.39, | 1.4) |
| rs16847084 | C., B. | 348/956 | 52/126 | 1.1 | (0.80, | 1.6) | 1.1 | (0.77, | 1.6) |
| C/T | E., B. | 190/417 | 18/37 | 1.1 | (0.57, | 2.0) | 1.1 | (0.57, | 2.0) |
| rs16847086 A/G | E., B. | 143/298 | 63/136 | 0.97 | (0.63, | 1.5) | 0.97 | (0.63, | 1.5) |
| rs16847105 | C., W. | 285/1095 | 21/89 | 0.91 | (0.55, | 1.5) | 0.99 | (0.56, | 1.7) |
| T/G | E., B. | 192/410 | 32/56 | 1.2 | (0.72, | 2.1) | 1.7 | (0.63, | 4.7) |
| rs17766549 | C., B. | 391/1053 | 25/89 | 0.76 | (0.48, | 1.2) | 0.75 | (0.46, | 1.2) |
| C/G | C., W. | 251/970 | 55/20 | 1.0 | (0.75, | 1.4) | 0.97 | (0.67, | 1.4) |
|  | E., B. | 190/411 | 20/45 | 0.96 | (0.52, | 1.8) | 0.96 | (0.52, | 1.8) |
| rs1844885 | C., B. | 127/360 | 265/688 | 1.1 | (0.85, | 1.4) | 1.1 | (0.83, | 1.4) |
| G/A | C., W. | 158/607 | 138/511 | 1.0 | (0.80, | 1.3) | 0.91 | (0.68, | 1.2) |
|  | E., B. | 80/181 | 124/277 | 1.0 | (0.71, | 1.4) | 1.3 | (0.64, | 2.7) |
|  | E., W. | 231/888 | 201/784 | 0.99 | (0.80, | 1.2) | 1.1 | (0.80, | 1.5) |
| rs1873590 A/G | E., B. | 175/402 | 21/32 | 1.5 | (0.75, | 3.0) | 1.5 | (0.75, | 3.0) |
| rs188812 A/T | C., B. | 307/827 | 47/131 | 0.97 | (0.68, | 1.4) | 1.0 | (0.71, | 1.5) |
| rs2131033 A/G | E., B. | 129/308 | 55/130 | 1.0 | (0.65, | 1.6) | 1.0 | (0.65, | 1.6) |
| rs222010 C/T | C., W. | 284/1093 | 22/57 | 1.5 | (0.89, | 2.5) | 1.5 | (0.86, | 2.6) |
| rs222016 A/G | C., W. | 259/960 | 45/164 | 1.0 | (0.71, | 1.5) | 0.96 | (0.65, | 1.4) |
| rs222023 G/A | E., B. | 121/300 | 61/116 | 1.3 | (0.84, | 2.0) | 2.1 | (0.58, | 7.4) |
| rs222035 | C., B. | 327/847 | 61/173 | 0.91 | (0.66, | 1.3) | 0.93 | (0.66, | 1.3) |
| T/G | E., B. | 165/350 | 37/88 | 0.94 | (0.58, | 1.5) | 2.0 | (0.83, | 4.8) |
| rs222043 G/A | E., B. | 170/350 | 16/50 | 0.66 | (0.33, | 1.3) | 0.38 | (0.13, | 1.1) |
| rs222054 C/G | C., B. | 375/987 | 29/87 | 0.88 | (0.57, | 1.4) | 0.85 | (0.53, | 1.4) |
|  | C., W. | 252/969 | 36/155 | 0.89 | (0.61, | 1.3) | 0.83 | (0.55, | 1.3) |
|  | E., B. | 207/418 | 11/28 | 0.79 | (0.39, | 1.6) | 0.79 | (0.39, | 1.6) |
|  | E., W. | 383/1490 | 53/160 | 1.3 | (0.93, | 1.8) | 0.81 | (0.47, | 1.4) |
| rs2276461 | C., B. | 342/932 | 28/82 | 0.93 | (0.60, | 1.5) | 0.91 | (0.57, | 1.5) |
| G/A | E., B. | 190/406 | 14/34 | 0.88 | (0.45, | 1.7) | 0.65 | (0.20, | 2.1) |
| rs2282679 | C., B. | 375/987 | 39/121 | 0.85 | (0.58, | 1.2) | 0.84 | (0.57, | 1.2) |
| T/G | C., W. | 215/830 | 93/344 | 1.0 | (0.79, | 1.4) | 1.0 | (0.74, | 1.4) |
|  | E., B. | 191/426 | 23/54 | 0.95 | (0.53, | 1.7) | 0.91 | (0.33, | 2.5) |
| rs35096193 | C., B. | 401/1063 | 25/83 | 0.80 | (0.50, | 1.3) | 0.91 | (0.56, | 1.5) |
| C/A | E., B. | 190/424 | 22/190 | 1.4 | (0.71, | 2.6) | 0.45 | (0.11, | 1.8) |
| rs3775152 C/A | E., W. | 436/1636 | 32/98 | 1.2 | (0.81, | 1.9) | 0.73 | (0.38, | 1.4) |
| rs3775154 | C., B. | 381/977 | 23/97* | 0.61 | (0.38, | 0.97)** | 0.66 | (0.40, | 1.1) |
| C/G | E., B. | 194/418 | 14/30 | 1.0 | (0.51, | 2.0) | 1.0 | (0.51, | 2.0) |
| rs423578 C/T | C., B. | 238/668 | 138/368 | 1.1 | (0.82, | 1.3) | 1.1 | (0.82, | 1.4) |
| rs423817 G/T | C., B. | 228/574 | 148/376 | 0.99 | (0.78, | 1.3) | 1.0 | (0.79, | 1.3) |
| rs4364228 | C., B. | 238/646 | 162/428 | 1.0 | (0.81, | 1.3) | 1.1 | (0.86, | 1.4) |
| A/G | C., W. | 284/1099 | 14/81 | 0.67 | (0.37, | 1.2) | 0.71 | (0.39, | 1.3) |
| rs4518212 | C., B. | 272/765 | 124/269* | 1.3 | (1.0, | 1.7)** | 1.3 | (1.0, | 1.7)** |
| A/G | C., W. | 212/898 | 64/250 | 1.1 | (0.79, | 1.5) | 0.97 | (0.69, | 1.4) |
| rs451857 | C., B. | 285/714 | 125/364 | 0.86 | (0.67, | 1.1) | 0.84 | (0.65, | 1.0) |
| C/G | C., W. | 90/386 | 214/762 | 1.2 | (0.91, | 1.6) | 1.2 | (0.91, | 1.7) |
| rs4588 | C., B. | 363/1012 | 41/102 | 1.1 | (0.76, | 1.6) | 1.2 | (0.79, | 1.8) |
| G/T | C., W. | 214/845 | 80/317 | 1.0 | (0.75, | 1.3) | 1.0 | (0.75, | 1.4) |
| rs55986203 A/G | E., B. | 175/368 | 39/74 | 1.1 | (0.69, | 1.8) | 1.1 | (0.69, | 1.8) |
| rs56139968 | C., W. | 280/1074 | 26/94 | 1.1 | (0.67, | 1.7) | 1.1 | (0.67, | 1.8) |
| T/A | E., B. | 182/378 | 30/68 | 0.92 | (0.54, | 1.6) | 1.4 | (0.43, | 4.4) |
| rs62302186 G/T | C., W. | 236/1011 | 62/157* | 1.7 | (1.2, | 2.3)*** | 1.9 | (1.3, | 2.7)**** |
| rs66588463 | C., B. | 378/992 | 20/58 | 0.90 | (0.54, | 1.5) | 0.93 | (0.55, | 1.6) |
| A/C | E., B. | 190/405 | 12/25 | 1.0 | (0.51, | 2.1) | 1.0 | (0.51, | 2.1) |
|  | E., W. | 407/1580 | 25/102 | 0.95 | (0.61, | 1.5) | 0.93 | (0.43, | 2.0) |
| rs67573830 | C., B. | 354/955 | 46/151 | 0.82 | (0.58, | 1.2) | 0.79 | (0.55, | 1.1) |
| T/C | C., W. | 283/1131 | 17/75 | 0.91 | (0.53, | 1.6) | 0.92 | (0.49, | 1.7) |
|  | E., B. | 181/390 | 25/66 | 0.82 | (0.48, | 1.4) | 1.1 | (0.34, | 3.7) |
| rs6823308 | C., B. | 233/614 | 155/416 | 0.98 | (0.77, | 1.2) | 1.0 | (0.80, | 1.3) |
| A/C | C., W. | 124/427 | 166/699 | 0.82 | (0.63, | 1.1) | 0.75 | (0.56, | 1.0)** |
| rs6830709 | C., B. | 337/908 | 57/180 | 0.85 | (0.62, | 1.2) | 0.81 | (0.57, | 1.1) |
| G/A | E., B. | 1722/375 | 40/81 | 1.1 | (0.71, | 1.6) | 0.39 | (0.14, | 1.1) |
| rs6837292 T/C | E., B. | 181/373 | 33/81 | 0.84 | (0.51, | 1.4) | 1.7 | (0.53, | 5.3) |
| rs6837549 G/T | E., B. | 124/257 | 84/171 | 1.0 | (0.70, | 1.5) | 1.0 | (0.70, | 1.5) |
| rs7041 | C., B. | 374/994 | 28/68 | 1.1 | (0.69, | 1.7) | 1.1 | (0.66, | 1.7) |
| A/C | C., W. | 195/740 | 81/332 | 0.93 | (0.69, | 1.2) | 0.90 | (0.66, | 1.2) |
|  | E., W. | 308/1175 | 134/451 | 1.1 | (0.90, | 1.4) | 1.1 | (0.77, | 1.6) |
| rs72648067 A/G | E., B. | 180/393 | 32/35* | 2.0 | (1.1, | 3.7)** | 2.0 | (1.1, | 3.7)** |
| rs72860546 C/T | E., B. | 198/420 | 16/40 | 0.85 | (0.44, | 1.6) | 0.85 | (0.44, | 1.6) |
| rs74706296 C/T | C., W. | 276/1087 | 26/91 | 1.1 | (0.71, | 1.8) | 0.97 | (0.57, | 1.7) |
| rs7691724 A/G | C., B. | 350/948 | 14/56 | 0.68 | (0.37, | 1.2) | 0.77 | (0.42, | 1.4) |
| rs7696078 | C., B. | 355/871 | 57/147 | 0.95 | (0.68, | 1.3) | 1.0 | (0.71, | 1.4) |
| T/A | E., B. | 172/338 | 26/78 | 0.66 | (0.39, | 1.1) | 0.41 | (0.17, | 1.0) |
| rs78123752 | C., B. | 143/943 | 32/143* | 0.56 | (0.38, | 0.84)*** | 0.59 | (0.38, | 0.89)** |
| T/G | E., B. | 196/394 | 22/54 | 0.82 | (0.47, | 1.4) | 1.8 | (0.62, | 5.3) |
| rs842872 | C., B. | 335/893 | 71/213 | 0.89 | (0.66, | 1.2) | 0.93 | (0.68, | 1.3) |
| G/A | C., W. | 143/544 | 149/604 | 0.94 | (0.73, | 1.2) | 0.85 | (0.64, | 1.1) |
| rs842877 | C., B. | 370/967 | 40/107 | 0.98 | (0.67, | 1.4) | 0.96 | (0.64, | 1.4) |
| C/T | C., W. | 220/859 | 76/271 | 1.1 | (0.82, | 1.5) | 1.1 | (0.78, | 1.5) |
|  | E., B. | 173/407 | 31/55 | 1.3 | (0.81, | 2.2) | 1.3 | (0.81, | 2.2) |
|  | E., W. | 329/1236 | 113/454 | 0.94 | (0.74, | 1.2) | 0.75 | (0.51, | 1.1) |
| rs842881 | C., W. | 279/1101 | 21/77 | 1.1 | (0.65, | 1.8) | 1.0 | (0.58, | 1.8) |
| A/G | E., W. | 448/1679 | 30/85 | 1.3 | (0.86, | 2.0) | 0.95 | (0.53, | 1.7) |
| rs842891 | C., B. | 307/822 | 83/220 | 1.0 | (0.76, | 1.3) | 1.0 | (0.74, | 1.4) |
| G/A | C., W. | 255/1042 | 45/132 | 1.4 | (0.97, | 2.0) | 1.5 | (1.0, | 2.3)** |
|  | E., B. | 171/346 | 35/110* | 0.64 | (0.39, | 1.1) | 0.50 | (0.19, | 4.0) |
|  | E., W. | 406/1509 | 56/209 | 1.0 | (0.73, | 1.4) | 1.1 | (0.72, | 1.7) |
| rs842991 | C., B. | 321/947 | 63/131* | 1.4 | (1.0, | 2.0)** | 1.5 | (1.0, | 2.0)** |
| A/G | E., B. | 176/395 | 28/47 | 1.3 | (0.77, | 2.3) | 1.3 | (0.77, | 2.3) |
| rs842992 C/T | E., B. | 177/363 | 27/63 | 0.88 | (0.51, | 1.5) | 0.88 | (0.51, | 1.5) |
| rs842998 G/C | E., B. | 206/437 | 18/29 | 1.3 | (0.62, | 2.8) | 6.6 | (0.32, | 133) |
| rs843010 | C., B. | 325/896 | 47/106 | 1.2 | (0.85, | 1.8) | 1.3 | (0.86, | 1.9) |
| T/C | C., W. | 276/1119 | 26/69 | 1.5 | (0.95, | 2.4) | 1.7 | (1.0, | 2.9)** |
|  | E., B. | 168/382 | 18/62 | 0.66 | (0.35, | 1.2) | 0.85 | (0.37, | 1.9) |
|  | E., W. | 421/1625 | 31/109 | 1.1 | (0.73, | 1.7) | 2.0 | (1.2, | 3.3)*** |
| rs962225 | C., W. | 157/733 | 103/301* | 1.6 | (1.2, | 2.1)*** | 1.9 | (1.4, | 2.6)**** |
| G/A | E., B. | 84/162 | 116/244 | 0.92 | (0.63, | 1.3) | 0.87 | (0.35, | 2.2) |
| rs962227 | C., W. | 203/835 | 93/273* | 1.4 | (1.1, | 1.9)** | 1.7 | (1.2, | 2.3)*** |
| G/A | E., B. | 89/202 | 101/238 | 0.96 | (0.66, | 1.4) | 0.93 | (0.43, | 2.0) |

*p <0.05 based on a chi-square test. **ORs and 95% CI that reach statistical significance at p<0.05. ***ORs and 95% CI that reach statistical significance at p<0.01. ****ORs and 95% CI that reach statistical significance at p<0.001.

*GC*, vitamin D binding protein; CI, confidence interval; C., B., CPP study, Black race; C., W., CPP study, White race; E., B., EVITA study, Black race; E., W., EVITA study, White race

a Groups not shown for each SNP did not pass quality control steps.

b Adjusted for 25(OH)D concentration, maternal age, smoking, body mass index, percent African ancestry (Black mothers only), site (CPP only), year of blood draw (EVITA only), and batch (EVITA only). Significance of the associations are given by symbols.

Supplemental Table 3: Association between minor alleles of *CYP27B1* variants and the risk of preeclampsia compared with major alleles by maternal race and study.^a^

| Variant name | Study, Race | Number of controls [minor/major alleles] | Number of cases [minor/major alleles] | Univariate Analysis  Odds Ratio (95%CI) | | | Multivariate Analysis  Odds Ratio (95%CI) ^b^ | | |
| --- | --- | --- | --- | --- | --- | --- | --- | --- | --- |
| rs1021469 G/A | E., B. | 171/ 379 | 43/85 | 1.1 | (0.71, | 1.8) | 1.7 | (0.58, | 5.0) |
| rs10431552 G/A | E., B. | 166/382 | 46/82 | 1.3 | (0.83, | 2.0) | 2.1 | (0.78, | 5.4) |
| rs1048691 C/T | E., B. | 135/308 | 69/124 | 1.3 | (0.84, | 1.9) | 1.3 | (0.84, | 1.9) |
| rs10877011 T/G | E., B. | 187/403 | 33/59 | 1.2 | (0.73, | 2.0) | 1.5 | (0.48, | 4.9) |
| rs10877012 | C., B. | 355/1004 | 47/118 | 1.1 | (0.79, | 1.6) | 1.2 | (0.81, | 1.7) |
| G/T | C., W. | 210/844 | 86/320 | 1.1 | (0.81, | 1.4) | 1.1 | (0.80, | 1.5) |
|  | E., B. | 186/399 | 24/59 | 0.87 | (0.52, | 1.5) | 0.87 | (0.52, | 1.5) |
| rs10877016 | C., B. | 330/833 | 56/149 | 1.0 | (0.72, | 1.4) | 1.1 | (0.81, | 1.6) |
| A/G | C., W. | 199/772 | 73/296 | 0.96 | (0.71, | 1.3) | 1.0 | (0.71, | 1.9) |
|  | E., B. | 173/383 | 25/57 | 0.97 | (0.55, | 1.7) | 0.97 | (0.55, | 1.7) |
| rs11172322 | C., W. | 280/1080 | 24/90 | 1.0 | (0.64, | 1.6) | 1.1 | (0.69, | 1.9) |
| T/C | E., B. | 168/375 | 34/61 | 1.2 | (0.73, | 2.1) | 0.99 | (0.26, | 3.7) |
|  | E., W. | 423/1522 | 35/150 | 0.84 | (0.57, | 1.2) | 0.59 | (0.29, | 1.2) |
| rs114001995 G/A | C., B. | 325/898 | 23/74 | 0.86 | (0.53, | 1.4) | 0.87 | (0.53, | 1.4) |
| rs11829917 G/A | C., W. | 190/782 | 62/254 | 1.0 | (0.73, | 1.4) | 1.0 | (0.71, | 1.5) |
| rs12318065 | C., B. | 340/892 | 50/138 | 0.95 | (0.67, | 1.3) | 0.97 | (0.67, | 1.4) |
| C/A | E., B. | 170/326 | 28/88* | 0.61 | (0.37, | 1.0) | 0.33 | (0.13, | 0.84)** |
| rs12368653 G/A | E., B. | 163/307 | 55/141 | 0.73 | (0.48, | 1.1) | 0.45 | (0.16, | 1.3) |
| rs12422249 | C., B. | 388/1023 | 14/61 | 0.61 | (0.33, | 1.1) | 58 | (0.31, | 1.1) |
| G/A | E., B. | 215/421 | 9/31 | 0.57 | (0.27, | 1.2) | 0.33 | (0.10, | 1.2) |
| rs12582311 | C., W. | 248/887 | 52/265* | 0.70 | (0.51, | 0.98)** | 0.70 | (0.48, | 1.0) |
| A/G | E., B. | 131/291 | 73/141 | 1.2 | (0.77, | 1.7) | 1.2 | (0.77, | 1.7) |
| rs17852479 | C., B. | 373/990 | 31/96 | 0.86 | (0.56, | 1.3) | 0.98 | (0.62, | 1.6) |
| C/A | E., B. | 189/410 | 25/70 | 0.77 | (0.43, | 1.4) | 0.44 | (0.16, | 1.2) |
|  | E., W. | 257/997 | 205/697 | 1.1 | (0.93, | 1.4) | 1.1 | (0.79, | 1.5) |
| rs2229103 T/C | E., B. | 208/445 | 12/33 | 0.78 | (0.40, | 1.5) | 0.32 | (0.13, | 0.78)** |
| rs2269720 | C., B. | 358/972 | 40/120 | 0.91 | (0.62, | 1.3) | 1.2 | (0.77, | 1.8) |
| T/C | C., W. | 198/788 | 84/340 | 0.98 | (0.74, | 1.3) | 0.97 | (0.71, | 1.3) |
|  | E., B. | 184/405 | 30/59 | 1.1 | (0.67, | 1.9) | 0.76 | (0.30, | 1.9) |
| rs2270777 | C., B. | 345/918 | 57/168 | 0.90 | (0.65, | 1.2) | 0.96 | (0.68, | 1.4) |
| C/T | C., W. | 168/658 | 124/488 | 1.0 | (0.77, | 1.3) | 1.0 | (0.76, | 1.4) |
|  | E., B. | 178/381 | 34/81 | 0.90 | (0.55, | 1.5) | 0.90 | (0.55, | 1.5) |
|  | E., W. | 266/1002 | 194/722 | 1.0 | (0.82, | 1.2) | 0.94 | (0.68, | 1.3) |
| rs238516 | C., W. | 156/597 | 132/501 | 1.0 | (0.78, | 1.3) | 1.1 | (0.80, | 1.4) |
| A/G | C., B. | 295/800 | 93/272 | 0.93 | (0.71, | 1.2) | 0.91 | (0.69, | 1.2) |
|  | E., B. | 144/317 | 66/129 | 1.1 | (0.76, | 1.7) | 1.1 | (0.76, | 1.7) |
| rs36227551 | C., B. | 354/924 | 58/178 | 0.85 | (0.62, | 1.2) | 0.91 | (0.65, | 1.3) |
| T/A | C., W. | 308/1164 | 14/64 | 0.83 | (0.46, | 1.5) | 0.81 | (0.43, | 1.5) |
|  | E., B. | 188/412 | 24/64 | 0.82 | (0.50, | 1.4) | 0.74 | (0.37, | 1.5) |
|  | E., W. | 458/1743 | 32/99 | 1.2 | (0.81, | 1.9) | 1.3 | (0.68, | 2.4) |
| rs3816896 C/T | C., B. | 335/866 | 67/192 | 0.90 | (0.66, | 1.2) | 0.97 | (0.70, | 1.3) |
| rs701006 A/G | E., B. | 119/237 | 65/185* | 0.70 | (0.46, | 1.1) | 0.70 | (0.46, | 1.1) |
| rs701007 | C., W. | 170/678 | 128/482 | 1.1 | (0.82, | 1.4) | 1.1 | (0.80, | 1.4) |
| C/A | E., B. | 169/342 | 47/124 | 0.77 | (0.50, | 1.2) | 0.45 | (0.22, | 0.90)** |
| rs701008 | C., B. | 354/960 | 54/158 | 0.93 | (0.66, | 1.3) | 1.1 | (0.74, | 1.5) |
| T/C | C., W. | 197/742 | 105/424 | 0.93 | (0.72, | 1.2) | 0.91 | (0.67, | 1.2) |
|  | E., B. | 183/383 | 35/81 | 0.90 | (0.57, | 1.4) | 1.0 | (0.39, | 2.5) |
|  | E., W. | 313/1091 | 151/649 | 0.81 | (0.65, | 1.0) | 0.84 | (0.60, | 1.2) |
| rs73338229 C/G | E., B. | 190/443 | 22/35 | 1.5 | (0.77, | 2.8) | 1.5 | (0.77, | 2.8) |
| rs73338231 | C., B. | 329/933 | 69/175 | 1.1 | (0.82, | 1.5) | 1.1 | (0.81, | 1.5) |
| A/G | E., B. | 191/413 | 29/51 | 1.2 | (0.73, | 2.1) | 1.2 | (0.73, | 2.1) |
| rs8176348 | C., B. | 325/899 | 71/185 | 1.1 | (0.79, | 1.4) | 1.0 | (0.74, | 1.4) |
| G/A | E., B. | 179/372 | 39/66 | 1.2 | (0.77, | 2.0) | 1.7 | (0.66, | 4.6) |

*^*^*p <0.05 based on a test for trend. **Betas and 95% CI that reach statistical significance at p<0.05. ***Betas and 95% CI that reach statistical significance at p<0.01. ****Betas and 95% CI that reach statistical significance at p<0.001.

*CYP27B1,* 1 alpha-hydroxylase; 25(OH)D, 25-hydroxyvitamin D; CI, confidence interval; C., B., CPP study, Black race; C., W., CPP study, White race; E., B., EVITA study, Black race; E., W., EVITA study, White race

a Groups not shown for each SNP did not pass quality control steps.

b Adjusted for 25(OH)D concentration, maternal age, smoking, body mass index, percent African ancestry (Black mothers only), site (CPP only), year of blood draw (EVITA only), and batch (EVITA only). Significance of the associations are given by symbols.
